# The economic costs of chronic wasting disease in the United States

**DOI:** 10.1371/journal.pone.0278366

**Published:** 2022-12-08

**Authors:** Scott J. Chiavacci

**Affiliations:** U.S. Geological Survey, Science and Decisions Center, Reston, VA, United States of America; Colorado State University College of Veterinary Medicine and Biomedical Sciences, UNITED STATES

## Abstract

Cervids are economically important to a wide range of stakeholders and rights holders in the United States. The continued expansion of chronic wasting disease (CWD), a fatal neurodegenerative disease affecting wild and farmed cervids, poses a direct and indirect threat to state and federal government agency operations and cervid related economic activity. However, the scale of this disease’s direct economic costs is largely unknown. I synthesized existing publicly available data and stakeholder-provided data to estimate CWD’s costs within the continental United States. Federal government agencies collectively spent over $284.1 million on CWD-related efforts between 2000 and 2021, with $203.6 million of this total being spent by the U.S. Department of Agriculture’s Animal and Plant Health Inspection Service. In fiscal year 2020, state natural resources agencies and state agriculture/animal health agencies spent over $25.5 million and $2.9 million, respectively, on CWD-related work. Natural resources agencies in states with known CWD cases spent over 8 times as much on CWD as agencies from states with no known cases. The farmed cervid industry spent at least $307,950 on CWD sampling in 2020, though a lack of available data prevented a complete assessment of costs to this industry. Based on limited data, CWD’s economic effects on the hunting industry (i.e., outfitters and guides, companies leasing land to cervid hunters), may be negligible at this time. Overall, however, the realized economic costs of CWD appear considerable, and it is likely that the number of stakeholders financially affected by this disease and regulations meant to stem its spread will continue to grow. By understanding the current economic impacts of CWD, we are better positioned to assess the costs and benefits of investments in management and research and to understand the magnitude of this disease’s broader societal impacts.

## Introduction

Cervids like white-tailed deer (*Odocoileus virginianus*), mule deer (*O*. *hemionus*), and elk (*Cervus canadensis*), are economically important to a wide range of stakeholders and rights holders in the United States. The continued expansion of chronic wasting disease (CWD), a fatal neurodegenerative disease affecting wild, farmed, and captive cervids (farmed and captive cervids are hereafter collectively referred to as ‘farmed cervids’), has the potential to directly and indirectly affect cervid related economic activity [1–3]. However, the economic costs of CWD remain largely unknown, despite it presently being detected in 30 states [4].

There are multiple potential costs associated with CWD. These include direct economic costs such as personnel time, sample processing, travel expenditures, materials (e.g., personal protective equipment), management activities (e.g., sharpshooting, culling farmed cervids), regulations enforcement, outreach, veterinary expenses, and reduced product sales. The stakeholders facing such costs include state natural resource, animal health, and federal agencies in addition to the farmed cervid and hunting industries. The disease may also have broader societal impacts such as changes in hunter behavior and associated economic activity as well as effects on hunter and non-hunter satisfaction (‘utility’) related to cervids. Estimating CWD’s direct economic costs is a critical first step in understanding the magnitude of this disease’s societal costs.

Deer are the most popular game animals in the United States. In 2016, 8.1 million hunters (70% of all hunters) pursued deer [5]. These same hunters contributed $20.9 billion to the U.S. gross domestic product and generated $5 billion in taxes through spending on travel, lodging, meals, equipment, animal processing, guiding, land access, and other amenities [6]. Elk hunters, though fewer in number than deer hunters, (0.7 million in 2016; [5]) tend to spend more per hunting trip than other cervid hunters and are often strong economic contributors at local and regional scales [7]. Elk also attract non-consumptive users like tourists and wildlife photographers to areas where elk can be easily viewed [8–10]. Since wild cervid hunting and viewing are often concentrated in rural locations [e.g., 11], these activities help bolster the economies of communities that may otherwise have limited economic opportunities. The few studies of how CWD has affected hunter participation and connected economic activities illustrate that hunter participation can measurably decline where CWD is initially detected [12–14]. Further, estimates of the potential annual economic impacts of CWD-caused declines in deer hunter participation are thought to be in the tens of millions of dollars for individual states [2, 3, 15]. The much-expanded range of CWD in the United States since these studies were done suggests the economic costs of the disease on a national scale may be substantial.

Beyond spending on hunting-related travel and expenditures, deer hunters drive the bulk of hunting license sales in the United States [5]. Because these license sales contribute significantly to natural resources agency budgets [16], deer hunting can have a strong influence on agency operations and the management of resources beyond deer [2]. Further, many of these agencies are already financially strained because of a decades long decline in hunter numbers [17]. CWD’s negative effect on deer hunter participation and license sales, even if lasting only several years [12–14, 18], may be exacerbating already thinned agency budgets. Furthermore, a partial census of agency spending on CWD indicated states are collectively spending at least $10 million annually to track, communicate about, and contain the disease [19]. Thus, aside from investing resources in managing CWD for ecological reasons, state agencies also have a financial incentive to stem its spread.

Stakeholders connected to the farmed cervid industry (e.g., cervid breeders, operators of hunting preserves, regulatory agencies) are another group likely being economically impacted by CWD [1, 2, 20]. Many states allow cervid farming and hunting [21], and as of 2017, the United States contained over 212,000 farmed deer and over 31,000 farmed elk [22]. These operations are estimated to contribute $7.9 billion annually to the U.S. economy [23]. However, the movement of cervids among properties has been suggested as a source of CWD transmission over large distances [24]. State and federal regulations have been instituted to monitor for and minimize the chances of CWD transmission among cervid farms and hunting preserves and between farmed and wild cervids. Such regulations include farmed herds being quarantined for several years or depopulated following CWD detection, double fencing requirements, and restrictions on movement of live animals or high-risk parts within and among states. Along with reduced demand for animal products, these regulations could be having an economic impact on farmed cervid operators [1, 20]. Agencies that regulate these farmed cervid operations may be engaged in more intensive herd monitoring and regulations enforcement in response to CWD. In states containing farmed cervid breeding or hunting, regulatory authority most often falls on animal health divisions within state departments of agriculture. Like natural resources agencies, state agriculture agencies are presumably also incurring costs (e.g., personnel time and other resources) because of CWD.

Lastly, despite federal agencies having been directly engaged in or having supported non-federal entities in CWD research, outreach, and management efforts for 20 years [25], the level of spending by the federal government in response to the disease has not been thoroughly quantified. The U.S. Department of Agriculture (USDA), for example, has not only provided financial support to state agencies for CWD-related work [16] and engaged in their own research [26], they have also provided indemnity payments to cervid farmers whose animals are killed to prevent the spread of CWD [27]. The U.S. Fish and Wildlife Service helps direct funding to state natural resources agencies via the Wildlife and Sport Fish Restoration program and further assists states with CWD surveillance activities. Additionally, agencies like the National Institutes of Health and the U.S. Geological Survey have been and remain actively involved in CWD research efforts [e.g., 28–30]. Quantifying how much the federal government has spent on CWD-related efforts can enable an assessment of the resources devoted to managing and understanding this disease and the outcomes of such efforts.

I sought to estimate the direct costs of CWD on stakeholders and industries connected to wild and farmed cervids in the United States. My objectives were to (a) identify the stakeholder groups and industries connected to wild or farmed cervids at risk of being financially affected by CWD, (b) synthesize existing data to establish a baseline realized economic cost of the disease, and (c) highlight what data gaps could be addressed to advance our understanding of CWD’s impacts on the U.S. economy and societal well-being.

## Materials and methods

From May 2020 through September 2021, I sought data on CWD-related costs from all states in the continental United States regardless of CWD detection status in farmed or wild cervids. I excluded Hawaii because CWD testing of hunter killed cervids has been discontinued, the state had only two cervid farms with an unknown number of animals as of 2017, testing of farmed cervids is not mandatory [22, 31], and the only wild cervid inhabiting the Hawaiian Islands is the non-native Axis deer (*Axis axis*), which is considered non-susceptible or at low risk for contracting CWD [32].

I used the peer-reviewed literature to develop a list of stakeholders at risk of being economically impacted by CWD and identified any published estimates of CWD’s realized economic costs. Although I identified several studies of CWD’s economic impacts within specific states, these studies provided only potential costs of the disease [2, 3, 15]. I therefore relied on publicly available data and data shared by stakeholders to estimate CWD’s realized economic costs. I did not administer standardized surveys or conduct formalized interviews, nor did I establish a target sample size of stakeholders to contact (except for state agencies regulating wild or captive cervids) or collect data from a random sample of stakeholders and sources. Rather, I aimed to collect as much existing data on CWD’s financial effects as possible to estimate the current realized economic costs of the disease and identify data gaps. To increase my sample size, I asked stakeholders I spoke with to share contact information for other individuals who may have data on CWD’s economic impacts (i.e., a snowball sampling approach [33]. When gathering data from state and federal government agencies, I strove to collect data from all agencies engaged in CWD-related work. I also sought data on CWD-caused changes in hunting license or cervid tag sales from state natural areas to assess if cervid hunter behavior may have changed due to CWD. I took this approach rather than surveying individual cervid hunters about changes in where or if they hunt in response to CWD because I lacked the resources to survey individual hunters. Similarly, to collect data on the financial impacts of CWD on non-government groups representing diverse stakeholders (e.g., cervid farmers, outfitters and guides), I contacted national- and state-level organizations representing these stakeholders, if such organizations existed and I was able to acquire point of contact information. Although not all cervid farmers and outfitters and guides may be part of larger organizations representing their industry, contacting individual stakeholders regarding if and how CWD has financially affected each of them was infeasible given the number of stakeholders comprising such groups, the large scope of my study, and limited resources. To understand if demand for private land leases used for cervid hunting changed due to CWD, I contacted timber companies who lease lands they manage to cervid hunters. Finally, because I included partial or approximated costs provided by government and non-government stakeholders in my cost estimates and may have inadvertently omitted stakeholders financially affected by the disease, cost estimates I present should be considered conservative.

I sought spending data by U.S. government agencies for CWD-related work of their own employees or federal funds provided to non-federal entities (e.g., university researchers, state natural resources agencies, Tribal Nations, cervid farmers). I included all federal spending on CWD through the end of fiscal year 2021 (30 September 2021) to estimate the collective amount spent by the U.S. government. I obtained data on federal funding of CWD research by searching the National Science Foundation’s (NSF) awards database and the National Institutes of Health’s (NIH) RePORTER database. I used the search terms “chronic wasting disease” and “CWD” to identify research projects that contained either of these search terms in project titles or abstracts. To remain conservative in estimating federal spending on CWD research, I excluded NSF- and NIH-funded studies whose stated focus was not exclusively on CWD (e.g., studies that explored CWD as one part of a larger study on multiple prion diseases). I collected additional U.S. government spending on CWD by extracting funding data from www.usaspending.gov, which tracks federal contracts, contract indefinite delivery vehicles, grants, direct payments, and other financial assistance awards (e.g., indemnity payments). I identified relevant data using the search terms “chronic wasting disease,” “CWD,” “indemnity,” and combinations of these terms. The search results returned both obligated and de-obligated (negative) award amounts; I did not exclude de-obligated award amounts (*n* = 3) to ensure accurate representation of government spending on CWD. I cross-referenced data collected via www.usaspending.gov with that from the NSF and NIH databases and removed awards identified in multiple databases to avoid double counting awards. Because data acquired via www.usaspending.gov go back to only 2008 (at which point 14 states had detected CWD in wild or farmed cervids), and all but two awards were delivered to non-federal entities, I sought additional data on what federal agencies spent in support of their own staff and CWD-related work in wild and farmed cervids. Agencies I contacted for additional data included the USDA Animal Plant Health Inspection Service (APHIS), U.S. Geological Survey (USGS), U.S. Fish and Wildlife Service (USFWS), and National Park Service (NPS). I contacted these agencies because of their direct engagement in CWD-related research, surveillance, and regulatory oversight. The APHIS provided annual appropriated funding amounts from 2003 through 2021; APHIS funding for CWD-related work can be found within the ‘Agriculture, Rural Development, Food and Drug Administration, and Related Agencies Appropriations Act’ for each fiscal year on the Appropriations and Budgets page of www.congress.gov. Since amounts provided by APHIS (and in the Appropriations Act documents) encompassed APHIS-directed funding awards I collected from www.usaspending.gov, and because I report individual awards in each fiscal year (S1 Table), I subtracted the summed amount of each fiscal year’s individual awards from the fiscal year amount APHIS was appropriated for CWD work (I report the difference between these two totals as “Additional APHIS spending” in S1 Table). The USGS provided data on appropriated funds devoted to CWD for fiscal years 2019, 2020, and 2021. CWD spending by USGS in 2019 and 2020 is reported in [34] and spending in 2021 is reported in [35]. To avoid overestimating USGS spending, I excluded funds listed on www.usaspending.gov that were awarded by the USGS during fiscal years 2020 or 2021. The USFWS confirmed that the awards data I extracted from www.usaspending.gov appeared to encompass their CWD-related expenditures through fiscal year 2021.

I contacted state natural resources agencies regarding their expenditures used specifically for CWD-related work during the most recent fiscal year (1 July– 30 June) for which complete data were available. In cases where state natural resources agencies did not regulate farmed cervids, I contacted state departments of agriculture or animal health regarding their CWD-related expenditures. Examples of agency expenditures I sought to collect included staff time and benefits (e.g., communications, enforcing regulations, travelling, sample collection and testing (including sample shipment), communication materials (public advertisements, brochures, signage), travel, materials and supplies (e.g., personal protective equipment, sample extraction tools), carcass removal and disposal, payments to taxidermists and meat processors for sample collection, culling (sharpshooting) or depopulation, and research (e.g., human dimensions surveys)). Though comparing the costs of addressing CWD before and after states detected the disease could illustrate how expenditures changed following detection, I sought state agency costs for a single fiscal year to minimize the influence of changes over time in how agencies tracked CWD expenditures, but also to minimize large imbalances in the time periods over which states were able to share data. The time between when agencies detected the disease in wild or farmed cervids and the year to which agency expenditures data applied was, on average, 15.7 years (standard deviation [SD] = 11.2 years, range: 2–53 years; *n* = 26) for state natural resources agencies and 16.6 years (SD = 4.9 years, range: 8–22 years; *n* = 9) for state agriculture agencies. Such long periods of time preclude a reliable comparison of within-agency changes in spending before and after disease detection because numerous agencies noted that the detail with which they tracked CWD expenditures changed over time. Although most natural resources and agriculture agencies reported expenditures for fiscal year 2020 (1 July 2019–30 June 2020; *n* = 48) some states reported expenditures for 2019 (*n* = 12) or 2021 (*n* = 4). To facilitate presentation and interpretation of data, I adjusted all state agency expenditures to 2020 U.S. dollars using the U.S. Bureau of Labor Statistics Consumer Price Index (CPI) Inflation Calculator. Because federal agencies sometimes supported CWD-related work by state agencies, I cross-referenced all federally funded work delivered to states in fiscal years 2019, 2020, and 2021 with data provided by state agencies for the same fiscal years. I deducted from state agency costs any funds listed on www.usaspending.gov as going to the state agency in the same fiscal year for which the state provided cost data. Lastly, I asked agencies in states with and without known CWD cases if they had data linking changes in hunting license or deer tag sales to CWD to understand if cervid hunters may be changing their hunting behavior (e.g., what state or region they hunt in) in response to the disease. Lastly, to assess patterns in state natural resources agency spending relative to time since CWD detection, I plotted the relationship between agency spending in fiscal year 2020 and years since CWD detection within each state. I also examined this relationship after controlling for the potential influence of deer hunter numbers in each state using the 2011 National Survey of Fishing, Hunting, and Wildlife-Associated Recreation [36], the last year state-level data for deer hunter numbers were presented in the survey; I divided CWD spending in each state by the number of deer hunters and plotted this against years since CWD detection.

I reached out to organizations representing farmed cervid owners to gather data on CWD’s financial impacts to their operations and, if available, data sources for industry-wide effects of CWD. I sought CWD-specific data on costs such as veterinary expenses, property maintenance (e.g., double fencing costs), sample collection and shipment, lost or gained market opportunities (reduced demand for products nationally or internationally, inability to sell animals to farms in certain states, increased demand for in-state animals or increased demand for hunts), and value of animals culled during depopulation relative to indemnity payments for those animals. I also sought data on CWD’s economic effects on industries financially supported by hunting. I identified or was given contact information for 6 timber companies that lease lands to cervid hunters and 1 company (Hunting Lease Network) that specializes in land leasing for hunting in 24 states. I contacted these companies to determine if CWD had caused changes in lease pricing or demand. Although timber land may be leased for non-cervid hunting recreation (e.g., turkey hunting), companies I spoke to noted that most leased land under their management is used for deer hunting in particular, with one company noting 95% of leases are for deer hunting. Therefore, I assumed any identified changes in lease prices or demand were driven primarily by changes in deer hunter behavior. I also identified and contacted 8 state-level associations representing outfitters and guides in 8 different western states (Colorado, Idaho, Montana, Nevada, New Mexico, Oregon, Washington, Wyoming) about any CWD-driven changes in outfitting and guiding demand and related business revenue within their respective states; CWD was not detected in Idaho, Nevada, Oregon, or Washington at the time I contacted organizations in these states. As with captive cervid farming, I contacted state-level organizations assuming they would have data or information about guides and outfitters experiencing CWD-caused changes in demand for their services.

## Results

### Federal government

**Fig 1:**
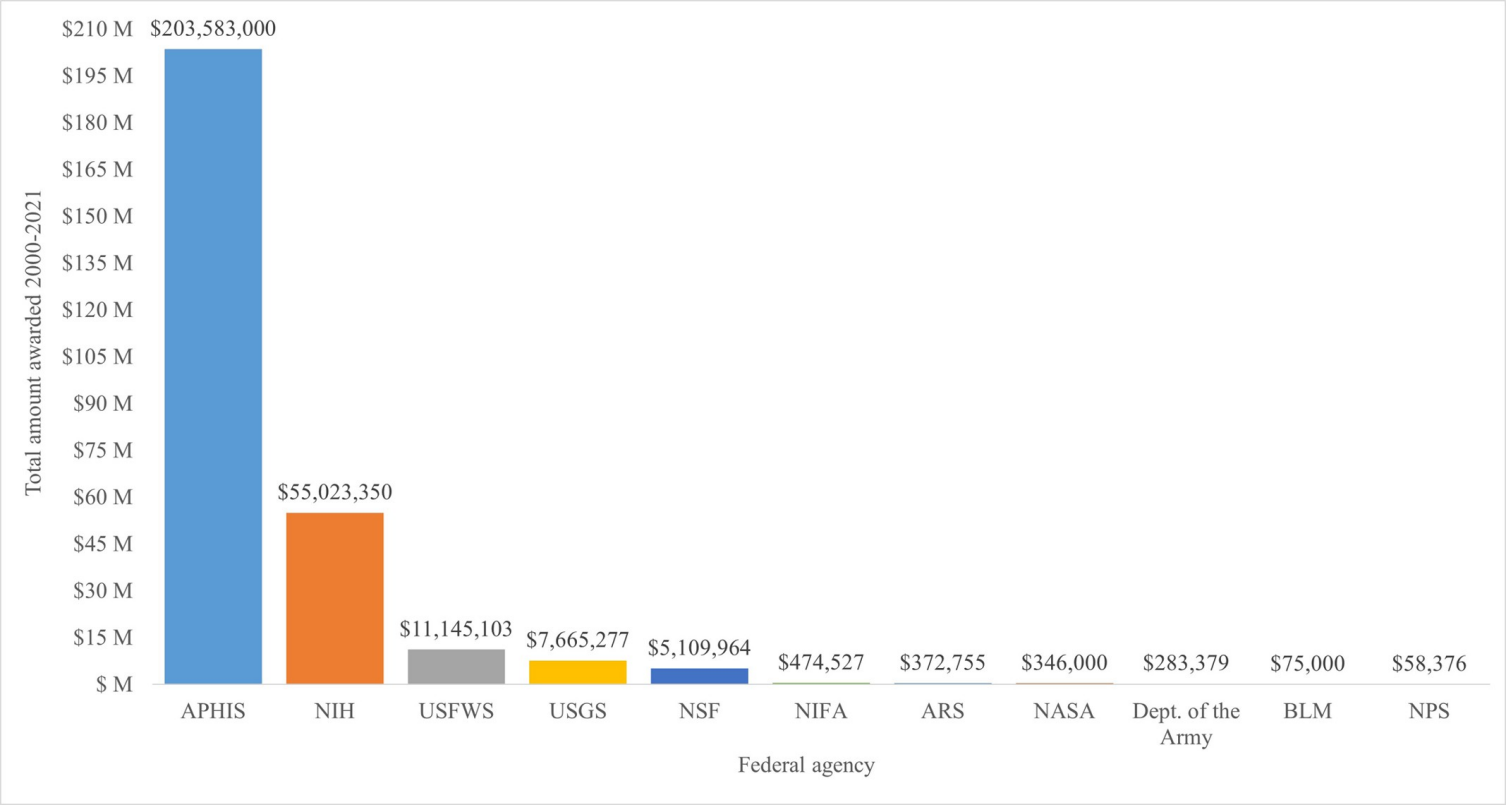
Spending by United States federal agencies related to chronic wasting disease, 2000–2021. APHIS = Animal and Plant Health Inspection Service, NIH = National Institutes of Health, USFWS = U.S. Fish and Wildlife Service, USGS = U.S. Geological Survey, NSF = National Science Foundation, NPS = National Park Service, NIFA = National Institute of Food and Agriculture, ARS = Agricultural Research Service, NASA = National Aeronautics and Space Administration, BLM = Bureau of Land Management.

Federal government agencies collectively spent at least $284.1 million from 2000 through fiscal year 2021 on CWD-related efforts such as research and surveillance, education and outreach, depopulation and indemnity, and agency operations (S1 Table and Fig 1). The APHIS spent markedly more on CWD than other agencies, with at least $16.5 million spent on indemnity payments to cervid farmers. Research awards given by the National Institutes of Health to support CWD research comprised the second largest amount of spending by a single federal agency and was approximately five times greater than USFWS, which spent the third largest amount among federal agencies.

### State natural resources and agriculture agencies

**Fig 2:**
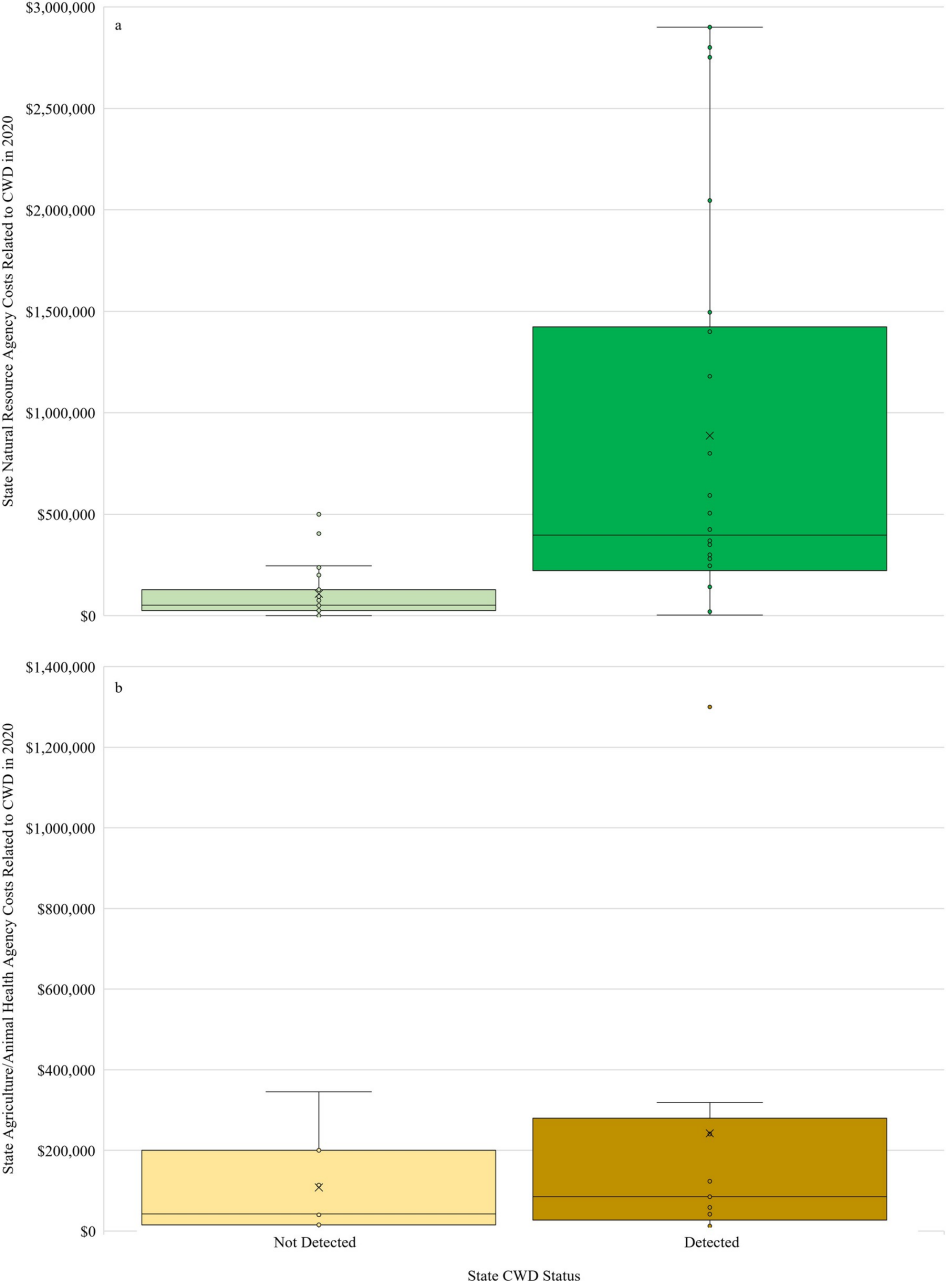
Distribution of reported state agency costs in fiscal year 2020 related to chronic wasting disease in states that have and have not detected the disease. Costs are displayed for (a) state natural resources agencies (*n* = 49) and (b) state agriculture/animal health agencies (*n* = 16) within the continental United States. The ‘x’ and horizontal lines inside each box represent the mean and median, respectively. The top and bottom of each box represent the median of the 3^rd^ and 1^st^ quartiles, respectively. ‘Whiskers’ extending above and below each box represent maximum and minimum costs not considered outliers.

**Fig 3:**
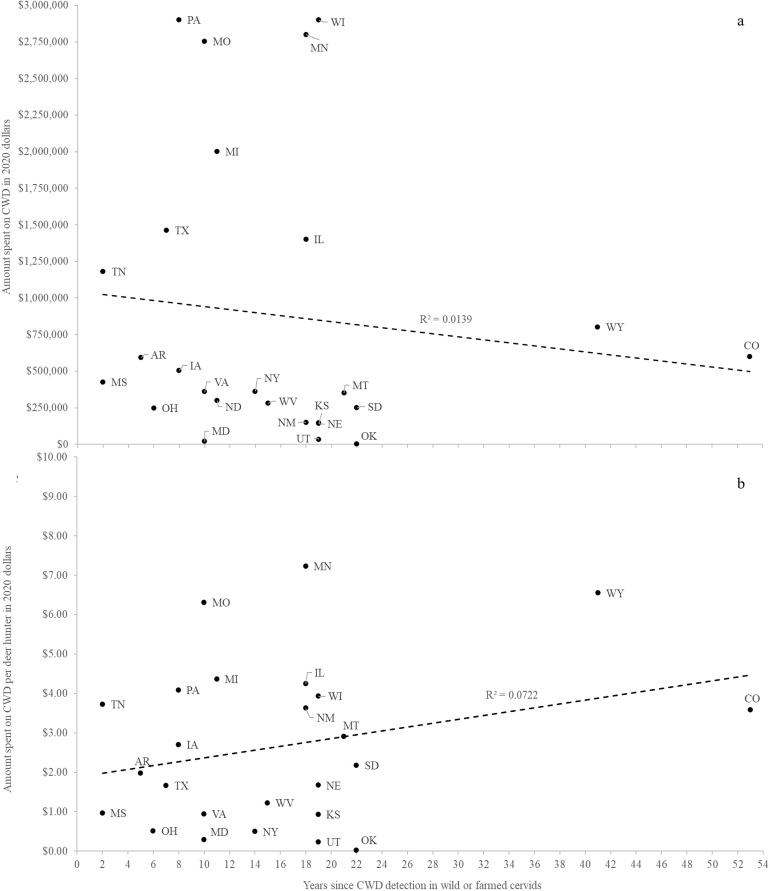
Relationship between time since chronic wasting disease (CWD) detection and money spent by state natural resources agencies on CWD-related work. Spending is displayed as (a) the relationship between time since CWD detection and spending and (b) the relationship between time since CWD detection and spending weighted by the number of deer hunters in each state. Deer hunter numbers were based on the 2011 National Survey of Fishing, Hunting, and Wildlife-Associated Recreation [35], the last year state-level data for deer hunter numbers were presented in the survey.

I obtained data on CWD-related costs from staff members of the 49 state natural resources agencies in the continental United States who managed wild cervids (9 of which noted that the costs they shared with me did not include every expenditure linked to the disease; Table 1). Ten of these agencies regulated both wild and farmed cervids, though four reported little or no costs related to farmed cervids because their states contained animals kept only as pets, on hobby farms, or in zoos or state regulations prohibited the containment, importation, or exportation of cervids. Collectively, state natural resources agencies in the continental United States spent at least $25.5 million on CWD-related work in fiscal year 2020 (Table 1). CWD costs averaged $521,261 per agency, though spending varied considerably among states (SD = $806,638, range: $0 –$2.9 million; *n* = 49). Annual spending also differed by CWD status–agencies from the 26 states with CWD detections in wild or farmed cervids as of the year for which they reported expenditures spent over eight times as much on average ($886,932 [SD = $981,800]) as agencies from the 23 states with no CWD detections ($107,894 [SD = $131,020]). However, variation in spending by agencies from states with CWD covered a much broader range than that of agencies from states without CWD (Fig 2A). Spending by agencies from states with known CWD cases appeared weakly related to time since CWD detection (Fig 3A), even when weighting agency spending by the number of deer hunters in each state to account for the possible influence of deer hunter abundance on CWD spending (Fig 3B). Eight natural resources agencies provided detailed breakdowns of expenditures, with 4 others providing minimally detailed breakdowns (i.e., ‘staff’ and ‘non-staff’ costs). All 12 of these agencies reported staff time, which made up an average of 50.1% (SD = 21.7%, range: 5.9–83.9%) of agency costs related to CWD during fiscal year 2020. Testing and lab costs were the second most reported expenditure (*n* = 8) and made up an average of 29.3% (SD = 23.2%, range: 6.3–78.4%) of agency costs. Equipment and supply expenditures were reported by 7 agencies and made up an average of 5.91% (SD = 6.2%, range: 1.5–19.1%) of costs. All other expenditure categories were reported by 4 or fewer agencies and are not presented here. Among agencies who discussed trends in hunting license or deer tag sales with me (*n* = 21 from states with CWD, *n* = 7 from states with no known CWD), all acknowledged knowing of no measurable effect of CWD on overall sales (one exception being the previously reported decline in Wisconsin’s license sales in 2002 and 2003 [12]). Thirteen agencies noted steady or increasing license sales, 10 of which were in states with CWD detections.

**Table 1:** Direct costs related to chronic wasting disease paid by state agencies and cervid farmers in the continental United States in 2020 dollars.

State	CWD Detected^a^	Natural Resources Agency Costs	Agriculture/Animal Health Agency Costs	Farmed Cervid Industry Costs^b^	Cervid Farms / Farmed Cervids^c^
Alabama	No	$42,500^d^	Not applicable^e^	Unknown	97 / 5,013
Alaska	No	$9,000^f^	$40,000	Unknown	6 / 260
Arizona	No	$83,500	Unknown	Unknown^g^	5 / 208
Arkansas	Yes	$592,218	Not applicable^e^	Unknown	13 / 446
California	No	$500,000	Not applicable^e^	Unknown^g^	8 / 256
Colorado	Yes	$600,000	Unknown	Unknown	46 / 1,336
Connecticut	No	$95,000	Not applicable^e^	Unknown^g^	15 / 168
Delaware	No	$32,500	Unknown	Unknown^g^	1 / unknown
Florida	No	$200,000^d^	Unknown^h^	$500	86 / 7,494
Georgia	No	$127,500	Unknown^j^	Unknown^g^	12 / 361
Idaho	No	$95,000	$15,000	$20,000	18 / 2,427
Illinois	Yes	$1,400,000^d^	Unknown	Unknown	83 / 1,543
Indiana	No	$127,856^j^	$113,863^d^^,^^j^	Unknown	143 / 3,888
Iowa	Yes	$505,000^d^	Unknown	Unknown	66 / 3,583
Kansas	Yes	$142,000	Unknown^h^	Unknown	35 / 2,957
Kentucky	No	$237,812^d^^,^^j^	$345,300^j^	Unknown	22 / 429
Louisiana	No	$404,425	$200,000	$1,200	82 / 3,733
Maine	No	$50,000	$42,448^k^	Unknown	50 / 7,837
Maryland	Yes	$20,000	----^i^	----^i^	0 / 0
Massachusetts	No	$500	Not applicable^e^	Unknown^g^	4 / 11
Michigan	Yes	$2,045,702^j^	Unknown	Unknown	178 / 16,842
Minnesota	Yes	$2,800,000	Unknown	Unknown	227 / 8,044
Mississippi	Yes	$425,000	Not applicable^e^	Unknown	44 / 1,611
Missouri	Yes	$2,752,403	Unknown	Unknown	70 / 4,163
Montana	Yes	$350,000	Unknown	Unknown	16 / 372
Nebraska	Yes	$149,336^j^	$42,000	Unknown	17 / 409
Nevada	No	$40,000	----^i^	----^i^	0 / 0
New Hampshire	No	$25,000	Unknown^h^	Unknown^g^	8 / 654
New Jersey	No	$51,142^k^	Not applicable^e^	Unknown^g^	9 / 172
New Mexico	Yes	$150,100	Not applicable^e^	Unknown	9 / 2,836
New York	Yes	$369,249^j^	$240,534	Unknown	107 / 7,437
North Carolina	No	$245,730	Unknown	Unknown	5 / 0
North Dakota	Yes	$300,000	$12,500	Unknown	26 / 2,256
Ohio	Yes	$246,000	Unknown	Unknown	215 / 6,223
Oklahoma	Yes	$2,500^d^	$85,000	Unknown	86 / 3,740
Oregon	No	$75,000	Not applicable^e^	Unknown^g^	11 / 283
Pennsylvania	Yes	$2,900,000	$1,300,000	Unknown	346 / 11,456
Rhode Island	No	$37,000	----^i^	----^i^	0 / 0
South Carolina	No	$0	----^i^	----^i^	10 / 387
South Dakota	Yes	$255,712^j^	$58,700^k^	Unknown	16 / 978
Tennessee	Yes	$1,179,776	Unknown^h^	Unknown	46 / 2,465
Texas	Yes	$1,495,718^j^	Not applicable^e^	Unknown	1,498 / 117,120
Utah	Yes	$31,300^d^^,^^k^	$123,320^d^^,^^k^	Unknown	21 / 733
Vermont	No	$1,000	Unknown	Unknown	4 / unknown
Virginia	Yes	$368,226^j^	----^i^	----^i^	2 / unknown
Washington	No	$1,100	$500	Unknown	12 / 523
West Virginia	Yes	$280,000^d^	$900^d^	$4,000	16 / 284
Wisconsin	Yes	$2,900,000	$318,852^j^	$246,608^j^	137 / 9,397
Wyoming	Yes	$800,000^d^	----^i^	----^i^	1 / unknown

^a^Denotes if CWD was detected in farmed or wild cervids as of the year for which CWD-related expenditures are reported. Note, four states (Alabama, Idaho, Louisiana, and North Carolina) detected CWD after data were collected

^b^All costs listed are for only sample extraction and testing costs paid by cervid farmers, as reported by state agencies that regulate farmed cervids

^c^Data are from the 2017 Census of Agriculture [37] and include deer and elk combined. These data may not correspond with the number of farms and animals in a state at the time of CWD cost data collection. ‘Unknown’ conveys that data on the number of cervid farms or cervids are not reported in the 2017 Census of Agriculture.

^d^Cost data provided by agency do not include all expenditures related to CWD work

^e^Farmed cervids are regulated by the state natural resources agency

^f^Cost was estimated at "less than $10,000 annually," so $9,000 was assumed to be costs for CWD-related work

^g^Costs to farmed cervid industry presumed small, if any, because of relatively small scale of industry, few animals, and regulations restricting animal importation and exportation

^h^Agency did not provide specific costs for farmed cervid oversight, but noted costs were minimal

^i^No farmed cervid industry cost data were collected

^j^Original values provided applied to fiscal year 2019 costs. These were converted to 2020 dollars using the U.S. Bureau of Labor Statistics CPI Inflation Calculator

^k^Original values provided applied to fiscal year 2021 costs. These were converted to 2020 dollars using the U.S. Bureau of Labor Statistics CPI Inflation Calculator

I contacted 28 state departments of agriculture that regulated farmed cervids in their states and inquired about their CWD-related expenditures. Sixteen of these agencies provided expenditures (4 of which noted the costs they shared did not include every expenditure linked to the disease), 2 did not provide expenditures data but noted CWD-related work carried very little cost to their agency, 5 responded but did not provide data, and 5 did not respond to my inquiries. CWD-related spending by state agencies overseeing farmed cervids in 2020 totaled over $2.9 million and averaged $183,682 per agency (SD = $317,629; Range: $500 –$1.3 million; *n* = 16). Agencies regulating farmed cervids in states with CWD detections as of the year for which they reported expenditures (*n* = 6) spent, on average, over double for CWD work ($242,423 [SD = $410,549]) compared to states with no known CWD ($108,159 [SD = $125,124]; *n* = 10; Fig 2B). However, this difference was primarily driven by a single agency’s large budget in a CWD positive state.

### Farmed cervid industry

I contacted 12 representatives from the farmed cervid industry for which I obtained contact information from other stakeholders. Of these, eight cervid farmers (one of whom held a leadership role in the North American Deer Farmers Association and six of whom held leadership roles in state-level organizations representing cervid farmers) discussed CWD’s economic impacts with me. All stated that CWD was negatively affecting their businesses and the broader industry, citing CWD regulations, especially movement restrictions, as a barrier to market opportunities and recruitment of new and retention of existing cervid farmers. However, the only quantitative statewide data I acquired that could be linked to CWD were costs paid by cervid farmers for sample collection and operation permits; all of these data were provided by state agriculture or animal health agencies who maintain state-level records of testing numbers and costs. Based on data from five states, CWD cost cervid farmers at least $307,950 in 2020, based on the processing of 630 samples at $40 each and 2447 samples at $100 each. In several states, regulatory agencies covered the costs of CWD sample collection and testing, reducing direct costs to cervid farmers; such costs are included in expenditures data reported for state agencies. Sample extraction and testing costs were most often reported as $40 –$45 per animal, but varied among and within states, with some cervid farmers reporting testing fees up to $150 per animal.

### Hunting industry

Of the eight state-level organizations representing outfitters and guides that I contacted three discussed CWD with me. Representatives from these organizations knew of no quantifiable economic effects of CWD on their industry within their respective states, though one guide noted a decrease in demand for guided mule deer hunts where CWD has caused a decline in the mule deer population. Of the six timber companies and one real estate company engaged in land leasing that I contacted, four discussed CWD costs with me (all timber companies). These four companies, who collectively manage 6.6 million hectares of land for timber production, knew of no current measurable effects of the disease on hunting lease prices or demand. Most of the lands managed by these companies are in southeastern states that have not yet detected CWD or have experienced only recent detections still concentrated in a small portion of a state. One timber company did, however, report the presence of vacant leases in a region where CWD was recently discovered, despite leases selling out in the rest of the state without known CWD cases.

## Discussion

Efforts to track, understand, and manage CWD are collectively costing state and federal agencies tens of millions of dollars annually. The disease’s costs to non-government stakeholders, however, are unclear and may remain challenging to reliably quantify. Nonetheless, the perpetual expansion of CWD into new areas of the United States [4] suggests the number of stakeholders affected and the costs to confront this disease will likely continue to increase. Although the extent to which CWD will accrue costs to the United States in the future is unknown, my study provides a baseline to which future assessments can compare and offers insights into how tracking the financial implications of this disease can be improved.

Natural resources agencies with known CWD cases in their states comprised the majority of state-level spending on CWD in 2020, highlighting the financial burden states can face once this disease is detected. It also illustrates that states tend to spend less on prevention than they do trying to manage the disease post-detection. A survey of state agencies suggests this pattern may result from a lack of adequate funding to confront CWD until the disease’s proximity warrants greater action [38]. Thus, states might benefit from analyzing the financial tradeoffs of investing in disease prevention versus long-term management efforts given how difficult CWD is to eliminate or contain [39, 40]. Notably, for agencies supported financially by hunting license sales, there appears to be little or no measurable effect of CWD on overall license purchases in most states at this time [also see 41–43]; however, the challenge in using license sales data alone to track the responses of hunters to CWD is that hunting licenses can often be used to hunt deer anywhere in a state (the exception being additional deer tags for specific regions), whereas states most often manage for and enact regulations related to CWD at the county level. Also, the liberalization of cervid harvest opportunities where CWD is detected (e.g., increased bag limits, removal of antler point restrictions) could attract new license purchasers (e.g., non-residents) who offset hunters who stopped hunting because of CWD. Further, when CWD is detected, it is typically on a small enough spatial scale that a limited number of hunters statewide are affected. Although evidence suggests a small proportion of hunters may change where or if they hunt cervids in response to CWD, these effects may diminish or even reverse themselves over time [13, 14]. Changes in cervid hunter behavior within a localized area could, however, have economic implications for the region, even if the change is only temporary. More studies surveying hunters following the detection of CWD where they hunt will help fill in gaps regarding hunter behavior and the potential economic implications of changes in hunter numbers or concentrations. Further, the decades long decline in hunter numbers across the United States adds urgency to the need to better track and understand such hunter responses to CWD. Nonetheless, cervid hunting’s popularity among hunters in the United States suggests CWD may continue to have only a small effect on hunter participation, barring CWD-caused declines in cervid hunting opportunities (e.g., cervid population declines) or the detection of CWD in humans. This continued hunter participation helps explain the lack of evidence for negative impacts of CWD on the hunting industry segments, such as outfitters and guides and land leasers. Although few industry representatives discussed the disease with me, it appears that if any economic impacts are occurring, they may be negligible at this time. But, as with other factors, the effects could appear minute on a large scale, but could carry serious financial implications for small businesses within or near CWD management zones (e.g., venison processors, taxidermists). Similarly, for state and federal government agencies whose budgets do not increase to help confront the expansion of CWD into new areas, the diversion of funds to support CWD-related work could reduce funding for other agency priorities and operational needs.

The diversity of federal agencies that have spent money on CWD exemplifies the scope of concern surrounding this disease and the United States government’s efforts to support CWD research, management, and education. The amount spent by the APHIS over the past two decades demonstrates the breadth of the agency’s investments in a variety of efforts, including indemnity payments to cervid farmers and supporting surveillance activities of state natural resources agencies and Tribal Nations. Similarly, the scale of research funding provided by the NIH highlights the agency’s efforts to support studies of CWD’s potential effects on human health. It is estimated that thousands of CWD positive animals are consumed annually in the United States [40] and this number could rise as CWD continues spreading. Given the remaining uncertainty about CWD’s capacity to infect humans and the scale of potential exposure, federal funding for human health research linked to the disease is likely to continue until the human health risks are better understood. The Chronic Wasting Disease Research and Management Act (H.R. 5608) currently introduced in the U.S. Congress, will, if passed, direct the USDA to deliver $70 million annually to states and Tribal Nations through fiscal year 2028. While this will increase federal spending on CWD, it will provide financial support to states and Tribal Nations that have been largely supporting their own CWD surveillance for years.

I was unable to fully assess the economic costs of CWD on the farmed cervid industry due to a lack of data, an issue noted in previous studies [2, 3, 20]. The only quantifiable impacts I identified were testing costs, though my estimate was based on data from only five states and is therefore an underestimate of the national cost of CWD to the industry. I did not extrapolate testing costs to states for which I did not have data because sample and testing costs vary among operations, not all farmed cervid owners are required to test animals, and in some states, costs are paid by state agencies. Testing costs appear, however, to be minor relative to the other costs of operating farmed cervid facilities (e.g., equipment, fencing, structural maintenance; [23]). In contrast, the closure and depopulation of CWD positive facilities as well as lost market opportunities and fewer industry participants resulting from CWD regulations (e.g., inability to import or export live cervids or cervid-derived products, costs of erecting double fencing) are likely to carry more substantial economic costs [20]. For example, although farmed cervid owners may be reimbursed for culled animals through the USDA’s Livestock Indemnity Program, the maximum amount paid per animal could be less than the animal’s market value, resulting in a financial loss to the owner. However, it is also possible some farmed cervid owners could indirectly benefit financially from CWD, such as when some farms are able to export animals to markets no longer available to competing farms that have been depopulated or face animal movement restrictions. Similarly, the closure of hunting preserves due to CWD could increase demand for hunts on preserves not affected by CWD (known as ‘leakage’ in economic terms). Further, farmed cervids are a primary source of income for some farmers and ranchers, while for others, cervids represent auxiliary sources of income or are kept as pets or maintained on hobby farms. Assessing these types of effects, and a range of other factors (see review in [20]), on the farmed cervid industry will continue to be challenging absent a concerted industry-wide effort to track and monetize CWD’s realized economic implications.

Concerns about the economic implications of CWD have existed for nearly 20 years [1, 2]. Yet, many unknowns remain about the scale of this disease’s economic costs. Although my study was relatively comprehensive, the costs I estimated should be considered a lower bound of a subset of direct costs and an indication of the potential magnitude of societal costs. For example, data on annual state agency spending for CWD was incomplete, as states varied in how thoroughly they tracked CWD-related expenditures and how much data they were able to share. Similarly, the data I used to estimate total federal agency spending is presumably less complete for years prior to 2008, the oldest year of data available on www.usaspending.gov, and because not all agencies were able to report funds spent to support internal operations related to CWD. I also obtained no quantitative data on CWD’s financial impacts on businesses linked to cervid hunting (e.g., outfitters and guides, timber companies leasing land to hunters). Although I spoke to representatives from each of these industries and all acknowledged knowing of no realized financial costs of the disease to industry stakeholders, unmeasured effects may exist. Other stakeholder groups that may also be facing unrecognized financial effects from CWD include taxidermists, meat processors, and property owners whose land value is tied to cervid hunting opportunities. Such entities could, in theory, experience (positive or negative) changes in demand for goods and services following CWD detection in their state, county, or other nearby market. It is unknown if such small-scale economic effects are occurring and how widely they might be experienced. However, the lack of a marked decline in cervid hunter participation due to CWD suggests the overall effects on most small businesses may be negligible at this time. Lastly, I did not assess CWD’s economic costs to American Indians or Alaska Natives who rely on cervids for food and materials and for whom cervids play a critical cultural role. It is also unknown what resources Tribes may be spending on CWD surveillance, outreach, and similar efforts independent of funds awarded to Tribal Nations by the U.S. government. Thus, the realized economic costs of CWD may be much greater than I estimated.

Based on my investigation, achieving a more complete understanding of CWD’s economic implications going forward would benefit from several changes in how CWD-related economic effects (positive or negative) are recorded. First, more thorough tracking of spending on CWD among state agencies can help fill in what are presumably major gaps in costs to these agencies (e.g., staff time). More complete data on resources being devoted by these agencies, as well as Tribal Nations, would facilitate more complete assessments of the costs of surveillance and management efforts relative to the benefits of these activities. An additional benefit of more complete state-level data is the ability to explore the potential costs and benefits of investing more money in preventing CWD from entering a state, given that costs to natural resources agencies increase considerably once the disease is detected in their states. Second, a concerted effort across the farmed cervid industry to estimate CWD’s financial effects would enable a needed assessment of the scale of this disease’s economic costs on one part of the private sector. Industry representatives noted the economic costs the disease is having on cervid farmers, and some data exist showing these costs [23], but none were able to provide quantitative data linking business costs or lost revenue directly to CWD. Third, given the limitations of using hunting license sales data to assess CWD-caused changes in cervid hunter participation, more surveys of how hunters are responding behaviorally to actual rather than hypothetical CWD outbreaks [e.g., 14, 39, 43, 44] would be useful. Such surveys could be designed to gather data on a range of realized impacts with economic implications, such as changes in hunting satisfaction, days spent afield, amount of venison discarded from CWD-positive animals, and spending on hunting equipment, travel, lodging, processing, and licenses and tags. Fourth, because CWD remains an expanding and evolving threat [4, 40], stakeholders currently unaffected or minimally affected by it (e.g., land leasers, guides and outfitters, taxidermists, meat processors, communities economically supported by elk viewing) could face serious economic consequences going forward. Thus, monitoring CWD-driven changes in pricing or demand for such goods and services would be prudent. Lastly, although I did not focus on or learn of any quantifiable benefits resulting from CWD, it is possible that factors such as investments in research, hiring of personnel to expand CWD-related capabilities, and increased spending by hunters who may travel outside of CWD management zones to hunt, could carry economic and societal benefits (e.g., a better understanding of prion disease transmissibility).

My study establishes a baseline cost of CWD in the United States and highlights factors needing further study that would help achieve a more holistic picture of this disease’s economic implications. The economic threat CWD poses is serious, and it is likely that the number of stakeholders financially affected by the disease or regulations meant to stem its spread will continue to grow. By understanding the current economic expenditures related to this disease, we are better positioned to assess the short- and long-term costs and benefits of investments in management and research.

## Supporting information

S1 Table Spending by the United States federal government related to chronic wasting disease research, surveillance, communication, indemnity payments, and depopulation of captive cervid facilities as of 30 September, 2021. Negative values represent a decrease in obligations (de-obligation) paid by the federal government due to, for example, changes in project costs or errors. Negative values were retained to reflect changes in obligated funding amounts. Awards with obligated amounts listed as $0 were excluded. Data are sorted first by ‘Awarding Agency, Sub-agency’ then by ‘Fiscal Year Funded’, ‘Data Source’, and ‘Award Description or Project Title’. Bolded ‘Award Amounts’ represent indemnity payments for culled captive cervids. APHIS = Animal and Plant Health Inspection Service, ARS = Agricultural Research Service, DOD = Department of Defense, DOI = Department of the Interior, HHS = Department of Health and Human Services, NASA = National Aeronautics and Space Administration, NIFA = National Institute of Food and Agriculture, NIH = National Institutes of Health, NPS = National Park Service, NSF = National Science Foundation, USDA = United States Department of Agriculture, USFWS = United States Fish and Wildlife Service, USGS = United States Geological Survey. Data source 1 = www.usaspending.gov; 2 = https://reporter.nih.gov/; 3 = https://nsf.gov/awardsearch/; 4 = https://www.congress.gov/help/appropriations-and-budget#:~:text=Appropriations%20and%20Budget%20Resources%20%7C%20Congress.gov%20%7C%20Library%20of%20Congress; 5 = https://d9-wret.s3.us-west-2.amazonaws.com/assets/palladium/production/s3fs-public/atoms/files/fy2021-usgs-budget-justification.pdf; 6 = https://www.appropriations.senate.gov/imo/media/doc/Division%20G%20-%20Interior%20Statement%20FY21.pdf; 7 = www.federalregister.gov/documents/2003/12/24/03-31543/chronic-wasting-disease-herd-certification-program-and-interstate-movement-of-captive-deer-and-elk. Any use of trade, firm, or product names is for descriptive purposes only and does not imply endorsement by the U.S. Government.
(DOCX)

## References

[1] Seidl AF, Koontz SR, Bruch M, Elder L. Economic implications of chronic wasting disease. Livestock and Wildlife Disease Report No. 7. Department of Agricultural and Resource Economics, Fort Collins, Colorado, USA; 2003.

[2] Bishop RC. The economic impacts of chronic wasting disease (CWD) in Wisconsin. Human Dimensions of Wildlife. 2004;3: 181–192.

[3] Seidl AF, Koontz SR. Potential economic impacts of chronic wasting disease in Colorado. Human Dimensions of Wildlife. 2004;3: 241–245.

[4] Richards B. Distribution of chronic wasting disease in North America (Updated). Available from: https://www.usgs.gov/media/images/distribution-chronic-wasting-disease-north-america-0.

[5] United States Fish and Wildlife Service, United States Department of the Interior, United States Department of Commerce, United States Census Bureau. 2016 National Survey of Fishing, Hunting, and Wildlife-Associated Recreation. FHW/16-NAT(RV). 2018.

[6] Southwick Associates. Hunting in America: An Economic Force for Conservation. Produced for the National Shooting Sports Foundation in partnership with the Association of Fish and Wildlife Agencies. 2012.

[7] Koontz L, Loomis JB. Economic importance of elk hunting in Jackson Hole, Wyoming. U.S. Geological Survey, Open-File Report 2005–1183, 1–24; 2005.

[8] Donovan G, Champ P. The economic benefits of elk viewing at the Jewell meadows wildlife area in Oregon. Human Dimensions of Wildlife. 2009;14: 51–60.

[9] Chapagain BP, Poudyal NC. Economic benefit of wildlife reintroduction: a case of elk hunting in Tennessee, USA. Journal of Environmental Management. 2020;269: 1–8. doi: 10.1016/j.jenvman.2020.110808 32561013

[10] Duda MD, Jones M, Beppler T, Bissell SJ, Center A, Criscione A, et al. Attitudes toward elk among EBCI members and visitors, and the economic impact of having elk on the Qualla Boundary. Responsive Management, Virginia, USA, 2020.

[11] Lauber TB, Brown TL. Deer hunting and deer hunting trends in New York State. Human Dimensions Research Unit Series No. 00–1. Department of Natural Resources, Cornell University, Ithaca, New York, USA; 2000.

[12] Heberlein TA. “Fire in the sistine chapel”: how Wisconsin responded to chronic wasting disease. Human Dimensions of Wildlife. 2004;9: 165–179.

[13] Haus JM, Eyler TB, Duda MD, Bowman JL. Hunter perceptions toward chronic wasting disease: implications for harvest and management. Wildlife Society Bulletin. 2017;41: 294–300.

[14] Holland AM, Haus JM, Eyler TB, Duda MD, Bowman JL. Revisiting hunter perceptions toward chronic wasting disease: changes in behavior over time. Animals. 2020;10: 1–10. doi: 10.3390/ani10020187 31978950 PMC7071074

[15] Menard J, Jensen K, English BC. Projected economic impacts of a chronic wasting disease (CWD) outbreak in Tennessee. Agri-Industry Modeling & Analysis Group Industry Brief; 2003.

[16] Gillin CM, Mawdsley JE. AFWA technical report on best management practices for surveillance, management and control of chronic wasting disease. Association of Fish and Wildlife Agencies, Washington, DC, USA. 2018. Goodman, L. A. 1961.

[17] Enck JW, Decker DJ, Brown TL. Status of hunter recruitment and retention in the United States. Wildlife Society Bulletin. 2000;28: 817–824.

[18] Miller CA. Deer hunter participation and chronic wasting disease in Illinois: an assessment at time zero. Human Dimensions of Wildlife. 2004;9:237–9.

[19] Quality Deer Management Association. QDMA’s Whitetail Report 2020. Bogart, Georgia, USA; 2020.

[20] Anderson A, Chomphosy WH. The impact of chronic wasting disease on the geographic distribution of the US captive cervid industry. Western Economics Forum. 2014;11–20.

[21] Adams KP, Murphy BP, Ross MD. Captive white-tailed deer industry—current status and growing threat. Wildlife Society Bulletin. 2016;40: 14–19.

[22] United States Department of Agriculture. 2017 Census of Agriculture. AC-17-A-51. 2019.

[23] Anderson DP, Outlaw JL, Earle M, Richardson JW. Economic impact of US deer breeding and hunting operations. College Station: Texas A&M University Agricultural and Food Policy Center. 2017. Pp. 17–24.

[24] Williams ES, Miller MW, Kreeger TJ, Kahn RH, Thorne ET. Chronic wasting disease of deer and elk: a review with recommendations for management. The Journal of Wildlife Management. 2002;66: 551–563.

[25] Angadjivand S, Crafton RE. Chronic wasting disease (CWD) and government response. Congressional Research Service. 2019; IF11213 7–5700. Available from https://sgp.fas.org/crs/misc/IF11213.pdf.

[26] United States Department of Agriculture, Animal and Plant Health Inspection Service. Accomplishments in chronic wasting disease research. 2020. Available from https://www.aphis.usda.gov/aphis/ourfocus/wildlifedamage/programs/nwrc/sa_spotlight/accomplishments_chronic_wasting_disease

[27] United States Department of Agriculture, Animal and Plant Health Inspection Service. Chronic wasting disease herd certification program and interstate movement of captive deer and elk. Federal Register. 2003; 68 FR 74513.

[28] Carlson CM, Hopkins MC, Nguyen NT, Richards BJ, Walsh DP, Walter WD. Chronic wasting disease—status, science, and management support by the U.S. Geological Survey Open-File Report 2017–1138; 2018. Available from https://pubs.er.usgs.gov/publication/ofr20171138.

[29] Race B, Williams K, Orrú CD, Hughson AG, Lubke L, Chesebro B. Lack of transmission of chronic wasting disease to cynomolgus macaques. Journal of Virology. 2018;92:e00550–18. doi: 10.1128/JVI.00550-18 29695429 PMC6026755

[30] Williams K, Hughson AG, Chesebro B, Race B. Inactivation of chronic wasting disease prions using sodium hypochlorite. PLOS ONE. 2019;14: e0223659. doi: 10.1371/journal.pone.0223659 31584997 PMC6777796

[31] Chronic Wasting Disease Alliance. Hawaii CWD Regulations. 2021 Oct [Cited 2021 November 1]. Available from: https://cwd-info.org/regulations/hawaii-cwd-regulations/.

[32] Buchholz MJ, Wright EA, Grisham BA, Bradley RD, Arsuffi TL, Conway WC. Characterization of the prion protein gene in axis deer (Axis axis) and implications for susceptibility to chronic wasting disease. Prion. 2021;15: 44–52. doi: 10.1080/19336896.2021.1910177 33834939 PMC8043172

[33] Goodman LA. Snowball sampling. The Annals of Mathematical Statistics. 1961;1: 148–70.

[34] United States Geological Survey. The United States Department of the Interior budget justifications and performance information: fiscal year 2021. 2020. Available from https://d9-wret.s3.us-west-2.amazonaws.com/assets/palladium/production/s3fs-public/atoms/files/fy2021-usgs-budget-justification.pdf

[35] United States Congress. Division G–Department of the Interior, Environment, and Related Agencies Appropriations Act, 2021. 2020. Available from https://www.appropriations.senate.gov/imo/media/doc/Division%20G%20-%20Interior%20Statement%20FY21.pdf

[36] United States Department of the Interior, United States Fish and Wildlife Service, and United States Department of Commerce, United States Census Bureau. 2011 National Survey of Fishing, Hunting, and Wildlife-Associated Recreation. 2011.

[37] United States Department of Agriculture National Agricultural Statistics Service. 2017 Census of Agriculture. 2019.

[38] Burroughs JP, Riley SJ, Taylor WW. Preparedness and capacity of agencies to manage chronic wasting disease. Human Dimensions of Wildlife. 2006;11: 227–228.

[39] Miller MW, Fischer JR. The first five (or more) decades of chronic wasting disease: lessons for the five decades to come. Transactions of the North American Wildlife and Natural Resources Conference. 2016;81: 110–120.

[40] Geist V, Clausen D, Crichton V, Rowledge D. The challenge of CWD: insidious and dire. Alliance for Public Wildlife; 2017.

[41] Zimmer NMP, Boxall PC, Adamowicz WL. The impacts of chronic wasting disease and its management on recreational hunters. Canadian Journal of Agricultural Economics. 2012;60: 71–92.

[42] Truong T, Adamowicz W, Boxall PC. Modelling the effect of chronic wasting disease on recreational hunting site choice preferences and choice set formation over time. Environmental and Resource Economics. 2018;70: 271–295.

[43] Erickson D, Reeling C, Lee JG. The effect of chronic wasting disease on resident deer hunting permit demand in Wisconsin. Animals. 2019;9: 1096. doi: 10.3390/ani9121096 31817847 PMC6941111

[44] Meeks A, Poudyal NC, Muller LI, Yoest C. Hunters concerns and intention to hunt in forested areas affected by wildlife disease. Forest Science. 2022;68: 85–94.

