# Supplementary material for: The economic costs of chronic wasting disease in the United States
Source: PLoS One. 2022 Dec 8;17(12):e0278366. doi: 10.1371/journal.pone.0278366 (PMC9731425; doi:10.1371/journal.pone.0278366)
Supplement: S1 Table — Negative values represent a decrease in obligations (de-obligation) paid by the federal government due to, for example, changes in project costs or errors. Negative values were retained to reflect changes in obligated funding amounts. Awards with obligated amounts listed as $0 were excluded. Data are sorted first by ‘Awarding Agency, Sub-agency’ then by ‘Fiscal Year Funded’, ‘Data Source’, and ‘Award Description or Project Title’. Bolded ‘Award Amounts’ represent indemnity payments for culled captive cervids. APHIS = Animal and Plant Health Inspection Service, ARS = Agricultural Research Service, DOD = Department of Defense, DOI = Department of the Interior, HHS = Department of Health and Human Services, NASA = National Aeronautics and Space Administration, NIFA = National Institute of Food and Agriculture, NIH = National Institutes of Health, NPS = National Park Service, NSF = National Science Foundation, USDA = United States Department of Agriculture, USFWS = United States Fish and Wildlife Service, USGS = United States Geological Survey. Data source 1 = www.usaspending.gov; 2 = https://reporter.nih.gov/; 3 = https://nsf.gov/awardsearch/; 4 = https://www.congress.gov/help/appropriations-and-budget#:~:text=Appropriations%20and%20Budget%20Resources%20%7C%20Congress.gov%20%7C%20Library%20of%20Congress; 5 = https://d9-wret.s3.us-west-2.amazonaws.com/assets/palladium/production/s3fs-public/atoms/files/fy2021-usgs-budget-justification.pdf; 6 = https://www.appropriations.senate.gov/imo/media/doc/Division%20G%20-%20Interior%20Statement%20FY21.pdf; 7 = www.federalregister.gov/documents/2003/12/24/03-31543/chronic-wasting-disease-herd-certification-program-and-interstate-movement-of-captive-deer-and-elk. Any use of trade, firm, or product names is for descriptive purposes only and does not imply endorsement by the U.S. Government. (DOCX) [file pone.0278366.s001.docx]

**S1 Table. Spending by the United States federal government on chronic wasting disease research, surveillance, management, communication, indemnity payments, and depopulation of farmed cervid facilities as of 30 September 2021.**

| Awarding Agency, Sub-agency | Total Obligated Award Amount | Recipient Name | Fiscal Year Funded | Award Description or Project Title | Award ID or Project Number | Data Source |
| --- | --- | --- | --- | --- | --- | --- |
| DOD, Dept. of the Army | $283,379 | THE REGENTS OF THE UNIVERSITY | 2003 | INVESTIGATIONS OF IMMUNIZATION STRATEGIES AGAINST CHRONIC WASTING DISEASE IN DEER AND ELK | DAMD170310320 | 1 |
| DOI, BLM | $75,000 | GAME AND FISH COMMISSION, WYOMING | 2021 | PREDATION AND CWD MANAGEMENT | L21AC10168 | 1 |
| DOI, NPS | $54,027 | COLORADO STATE UNIVERSITY | 2009 | H2370094000, J2340090033 CHRONIC WASTING DISEASE AT ROMO NP | J2340090033 | 1 |
| DOI, NPS | $6,609 | COLORADO STATE UNIVERSITY | 2010 | EFFECTS OF CHRONIC WASTING DISEASE ON ELK POPULATIONS | P10AC00655 | 1 |
| DOI, NPS | -$2,260 | COLORADO STATE UNIVERSITY | 2011 | INTEGRATE AND DEVELOP FIELD AND LAB RESEARCH PROGRAMS ON CHRONIC WASTING DISEASE | P11AC91352 | 1 |
| DOI, USFWS | $3,216 | COLORADO STATE UNIVERSITY | 2007 | ANIMAL SAMPLES BEING TESTED BY LAB FOR CHRONIC WASTING DISEASE. | INF615408M300 | 1 |
| DOI, USFWS | $55,000 | KANSAS STATE UNIVERSITY | 2007 | CHRONIC WASTING DISEASE - WHITETAIL DEER - KANSAS | 601817G287 | 1 |
| DOI, USFWS | -$3,199 | KANSAS STATE UNIVERSITY | 2008 | CHRONIC WASTING DISEASE - WHITETAIL DEER - KANSAS | F07AP00041 | 1 |
| DOI, USFWS | $9,500 | GAME AND FISH COMMISSION, WYOMING | 2008 | CHRONIC WASTING DISEASE REPORT | INF615508M306 | 1 |
| DOI, USFWS | $460,004 | ILLINOIS DEPARTMENT OF NATURAL RESOURCES | 2008 | WILDLIFE AND CHRONIC WASTING DISEASE SURVEILLANCE | IL-W-146-R-4 | 1 |
| DOI, USFWS | $10,000 | GAME AND FISH COMMISSION, WYOMING | 2009 | CHRONIC WASTING DISEASE (CWD) TECHNICIAN TO COMPLETE CWD REPORT | INF65521AP009 | 1 |
| DOI, USFWS | $10,000 | FISH, WILDLIFE AND PARKS, MONTANA DEPARTMENT OF | 2009 | CHRONIC WASTING DISEASE REPORT | INF655219P105 | 1 |
| DOI, USFWS | $21,300 | GAME AND FISH COMMISSION, WYOMING | 2010 | CHRONIC WASTING DISEASE MONITORING W/REPORT | INF65521AP054 | 1 |
| DOI, USFWS | $10,000 | GAME AND FISH COMMISSION, WYOMING | 2010 | CHRONIC WASTING DISEASE REPORT | INF655219P106 | 1 |
| DOI, USFWS | $16,960 | ERIK OSNAS ECOLOGICAL CONSULTING | 2010 | ENVIRONMENTAL STUDY OF CHRONIC WASTING DISEASE EFFECTS | INF60181AP037 | 1 |
| DOI, USFWS | $21,634 | GAME AND FISH COMMISSION, WYOMING | 2011 | CHRONIC WASTING DISEASE SURVEY AND REPORT, WYOMING | INF11PX04829 | 1 |
| DOI, USFWS | $42,000 | NATURAL RESOURCES, ILLINOIS DEPARTM ENT OF | 2011 | WILDLIFE AND CHRONIC WASTING DISEASE SURVEILLANCE | F11AF00159 | 1 |
| DOI, USFWS | $24,164 | GAME AND FISH COMMISSION, WYOMING | 2012 | 2012/13 CWD SURVEILLANCE OF JACKSON ELK HERD | INF12PX02987 | 1 |
| DOI, USFWS | $3,822 | STERLING COLLEGE | 2012 | QUIVIRA NWR DEER CHRONIC WASTING DISEASE MONITORING | F12AP00968 | 1 |
| DOI, USFWS | $1,991 | COLORADO STATE UNIVERSITY | 2012 | WHO - BPA CALL - CWD TESTING | INF12PB00135 | 1 |
| DOI, USFWS | $316,997 | ILLINOIS DEPT NATURAL RESRCS | 2012 | WILDLIFE AND CHRONIC WASTING DISEASE SURVEILLANCE | F12AF00386 | 1 |
| DOI, USFWS | $24,800 | GAME AND FISH COMMISSION, WYOMING | 2013 | CWD SURVEILLANCE OF JACKSON ELK HERD | INF13PX01331 | 1 |
| DOI, USFWS | $317,000 | NATURAL RESOURCES, ILLINOIS DEPARTM NT OF | 2013 | WILDLIFE AND CHRONIC WASTING DISEASE SURVEILLANCE, W-146-R-9 | F13AF00431 | 1 |
| DOI, USFWS | $82,550 | NATURAL RESOURCES, UTAH DEPARTMENT OF | 2014 | CHRONIC WASTING DISEASE SAMPLING | F15AP00063 | 1 |
| DOI, USFWS | $8,536 | WILDLIFE CONSERVATION, OKLAHOMA DEPARTMENT OF | 2014 | CHRONIC WASTING DISEASE SURVEILLANCE IN OKLAHOMA | F14AP00913 | 1 |
| DOI, USFWS | $63,771 | GAME AND FISH COMMISSION, WYOMING | 2014 | CHRONIC WASTING DISEASE SURVEILLANCE JACKSON ELK HERD | F14AP00980 | 1 |
| DOI, USFWS | $22,799 | NATURAL RESOURCES, IOWA DEPARTMENT OF | 2014 | CWD MONITORING SAVANNA DISTRICT | INF14PX01821 | 1 |
| DOI, USFWS | $42,420 | WILDLIFE, PARKS AND TOURISM, KANSAS DEPARTME | 2014 | CWD SURVEILLANCE EXPANSION AND ENHANCED SURVEILLANCE USING PRION DETECTION | F14AP00995 | 1 |
| DOI, USFWS | $26,265 | ENERGY & ENVIRONMENTAL PROTECTION, CONNECTICU | 2014 | DETERMINATION OF CHRONIC WASTING DISEASE (CWD) WITHIN DEER POPULATION OF THE STATE OF CONNECTICUT | F14AC01156 | 1 |
| DOI, USFWS | $317,000 | NATURAL RESOURCES, ILLINOIS DEPARTM ENT OF | 2014 | WILDLIFE AND CHRONIC WASTING DISEASE SURVEILLANCE, W-146-R-10 | F14AF00381 | 1 |
| DOI, USFWS | $33,750 | COLORADO STATE UNIVERSITY | 2015 | Analyze tissue specimens from deer and elk for Chronic Wasting Disease (CWD). | F15AF00514 | 1 |
| DOI, USFWS | $42,167 | COLORADO STATE UNIVERSITY | 2015 | DEVELOPING A PROACTIVE FRAMEWORK FOR ADAPTIVE MANAGEMENT OF CHRONIC WASTING DISEASE ON THE NATIONAL ELK REFUGE | F14AP00934 | 1 |
| DOI, USFWS | $64,715 | NATURAL RESOURCES, ILLINOIS DEPARTM ENT OF | 2015 | EVALUATING GENE EXPRESSION IN CWD-INFECTED WHITE-TAILED DEER, W-178-R-1 | F14AF00916 | 1 |
| DOI, USFWS | $201 | COLORADO STATE UNIVERSITY | 2015 | PROVIDE CHRONIC WASTING DISEASE (CWD) DIAGNOSTIC SERVICES | INF15PB00217 | 1 |
| DOI, USFWS | $158,371 | TEXAS PARKS AND WILDLIFE DEPARTMENT | 2015 | TX W-169-R-1 DNA TECHNOLOGIES TO GUIDE MANAGEMENT FOR CHRONIC WASTING DISEASE | F15AF01059 | 1 |
| DOI, USFWS | $357,994 | NATURAL RESOURCES, ILLINOIS DEPARTM ENT OF | 2015 | WILDLIFE AND CHRONIC WASTING DISEASE SURVEILLANCE, W-146-R-1 | F15AF00320 | 1 |
| DOI, USFWS | $214,838 | SOUTHERN ILLINOIS UNIVERSITY | 2016 | COOPERATIVE FOREST WILDLIFE RESEARCH ILLINOIS DEER INVESTIGATIONS | F16AF00493 | 1 |
| DOI, USFWS | $18,113 | ENERGY & ENVIRONMENTAL PROTECTION, CONNECTICU | 2016 | DETERMINE THE PRESENCE OR ABSENCE OF CHRONIC WASTING DISEASE IN THE DEER POPULATION OF THE STATE OF CONNECTICUT | F16AC00194 | 1 |
| DOI, USFWS | $357,996 | NATURAL RESOURCES, ILLINOIS DEPARTMENT OF | 2016 | WILDLIFE AND CHRONIC WASTING DISEASE SURVEILLANCE | F16AF00383 | 1 |
| DOI, USFWS | $218,596 | SOUTHERN ILLINOIS UNIVERSITY | 2017 | ILLINOIS DEER INVESTIGATIONS | F17AF00295 | 1 |
| DOI, USFWS | $31,646 | MISSISSIPPI STATE UNIVERSITY | 2017 | Modeling Population Status and Hunting Opportunities in Deer Herds Affected by Chronic Wasting Disease | F17AF01211 | 1 |
| DOI, USFWS | $406,643 | NATURAL RESOURCES, ILLINOIS DEPARTMENT OF | 2017 | WILDLIFE AND CHRONIC WASTING DISEASE SURVEILLANCE | F17AF00462 | 1 |
| DOI, USFWS | $67,969 | GAME AND FISH COMMISSION, WYOMING | 2018 | ASSESSING HARVEST MANGEMENT INFLUENCES ON CHRONIC WASTING DISEASE TRENDS IN THE WEST | F18AP00177 | 1 |
| DOI, USFWS | $26,974 | NATURAL RESOURCES, IOWA DEPARTMENT OF | 2018 | CHRONIC WASTING DISEASE MONITORING AND MANAGEMENT FOR FWS UPPER MISS. RIVER MCGREGOR DIST. | 140F0318P0060 | 1 |
| DOI, USFWS | $32,400 | COLORADO STATE UNIVERSITY | 2018 | CHRONIC WASTING DISEASE TESTING | INF17PD00298 | 1 |
| DOI, USFWS | $411,144 | NATURAL RESOURCES, ILLINOIS DEPARTMENT OF | 2018 | IL WILDLIFE AND CHRONIC WASTING DISEASE SURVEILLANCE W-146-R-14 | F18AF00406 | 1 |
| DOI, USFWS | $126,600 | WILDLIFE RESOURCES AGENCY, TENNESSEE | 2018 | TN-W-CWD STORAGE FREEZERS  GRANT IS TO BUY FREEZERS TO STORE DEER TO TEST FOR CWD | F18AF00503 | 1 |
| DOI, USFWS | $110,424 | UNIVERSITY OF TENNESSEE | 2019 | Chronic Wasting Disease Study | F19AF00465 | 1 |
| DOI, USFWS | $150,000 | COLORADO STATE UNIVERSITY | 2019 | CHRONIC WASTING DISEASE TESTING | 140F0619C0033 | 1 |
| DOI, USFWS | $62,031 | TEXAS STATE UNIVERSITY | 2019 | Public Perceptions of Chronic Wasting Disease in Western Texas | F19AF00901 | 1 |
| DOI, USFWS | $907,378 | WILDLIFE, PARKS AND TOURISM, KANSAS DEPARTMENT OF | 2019 | SPATIAL EPIDEMIOLOGY OF CHRONIC WASTING DISEASE | F19AF00655 | 1 |
| DOI, USFWS | $150,000 | Tennessee Department Of Agriculture | 2019 | To establish a new CWD testing center at Kord Animal Health Diagnostic Laboratory. | F19AF00532 | 1 |
| DOI, USFWS | $23,000 | NEBRASKA GAME AND PARKS COMMISSION | 2019 | TO MONITOR FOR CWD PREVALENCE DURING THE 2019 NINE-DAY FIREARM DEER SEASON IN THE PLAINS AND PINE RIDGE DEER MANAGEMENT UNITS BY COLLECTING RETROPHARYNGEAL LYMPH NODES AT ALL MAJOR FIREARM SEASON CHECK STATIONS IN THOSE TWO UNITS. | F20AC00184 | 1 |
| DOI, USFWS | $463,116 | NATURAL RESOURCES, ILLINOIS DEPARTMENT OF | 2019 | WILDLIFE AND CHRONIC WASTING DISEASE SURVEILLANCE, W-146-R-15 | F19AF00378 | 1 |
| DOI, USFWS | $46,000 | UNIVERSITY OF WYOMING | 2020 | Chronic Wasting Disease Collaborative Process | F19AF00668 | 1 |
| DOI, USFWS | $99,000 | MICHIGAN DEPARTMENT OF NATURAL RESOURCES | 2020 | COLLABORATIVE NETWORK-BASED TOO FOR IMPROVED CWD MANAGEMENT IN N.A. | F20AP00124 | 1 |
| DOI, USFWS | $30,000 | HDNR CONSULTING, LLC | 2020 | CO-NRPC-CHRONIC WASTING DISEASE IN ND | 140F0620C0035 | 1 |
| DOI, USFWS | $60,138 | COLORADO STATE UNIVERSITY | 2020 | CSU: CHARACTERIZING AND MAPPING CHRONIC WASTING DISEASE PRION STRAINS ACROSS THE UNITED STATES | F20AP00153 | 1 |
| DOI, USFWS | $133,843 | MISSISSIPPI STATE UNIVERSITY | 2020 | Funded project will investigate relationships between deer population density and Chronic Wasting Disease (CWD). | F20AF10434 | 1 |
| DOI, USFWS | $636,205 | NATURAL RESOURCES, ILLINOIS DEPARTMENT OF | 2020 | IL WILDLIFE AND CHRONIC WASTING DISEASE SURVEILLANCE W-146-R-16 | F20AF00292 | 1 |
| DOI, USFWS | $199,772 | GRAND PORTAGE RESERVATION TRIBAL COUNCIL | 2020 | MN CHRONIC WASTING DISEASE SURVEILLANCE IN MINNESOTA INDIAN COUNTRY U-NA | F20AP00240 | 1 |
| DOI, USFWS | $99,225 | WILDLIFE MANAGEMENT INSTITUTE, INCORPORATED | 2020 | NATIONAL COORDINATION AND TECHNICAL ASSISTANCE FOR THE PREVENTION, SURVEILLANCE, AND MANAGEMENT OF CHRONIC WASTING DISEASE (CWD) | F20AP00174 | 1 |
| DOI, USFWS | $161,246 | TEXAS PARKS AND WILDLIFE DEPARTMENT | 2020 | TX W-210-R-1: VALIDATION OF GENOMIC PREDICTIONS FOR DIFFERENTIAL SUSCEPTIBILITY TO CHRONIC WASTING DISEASE IN FREE-RANGING WHITE-TAILED DEER (ODOCOILEUS VIRGIANUS) | F21AF00238 | 1 |
| DOI, USFWS | $160,120 | NATURAL RESOURCES, INDIANA DEPARTMENT OF | 2020 | W-48-R-04 MITIGATING SPREAD OF CHRONIC WASTING DISEASE THROUGH AN ECOLOGICAL TRAP | F20AF10944 | 1 |
| DOI, USFWS | $138,697 | MICHIGAN DEPARTMENT OF NATURAL RESOURCES | 2020 | WSFR: A NOVEL GENETIC RESOURCE FOR CWD | F20AP00143 | 1 |
| DOI, USFWS | $99,741 | UNIVERSITY OF GEORGIA | 2020 | WSFR:UGA EXPLORING THE POTENTIAL FOR IN UTERO TRANSMISSION OF CWD PRIONS IN FREERANGING WHITE-TAILED DEER | F20AP00172 | 1 |
| DOI, USFWS | $125,778 | CALIFORNIA DEPARTMENT OF FISH AND WILDLIFE | 2021 | CHRONIC WASTING DISEASE SURVEILLANCE, EDUCATION AND OUTREACH | F21AF01594 | 1 |
| DOI, USFWS | $636,342 | NATURAL RESOURCES, ILLINOIS DEPARTMENT OF | 2021 | IL- WILDLIFE AND CHRONIC WASTING DISEASE SURVEILLANCE, W-146-R-17 | F21AF01411 | 1 |
| DOI, USFWS | $105,000 | NEBRASKA GAME AND PARKS COMMISSION | 2021 | NE W-156-R-1 INVESTIGATION OF THE FATE AND TRANSPORT OF CHRONIC WASTING DISEASE PRIONS AFTER LANDFILLING OR BURIAL | F21AF03422 | 1 |
| DOI, USFWS | $225,000 | NATURAL RESOURCES WEST VA DIV | 2021 | ONLINE PLATFORM FOR CHRONIC WASTING DISEASE DATA SHARING MANAGEMENT IN NORTH AMERICA | F21AP00619 | 1 |
| DOI, USFWS | $336,897 | GAME AND FISH, NORTH DAKOTA DEPARTMENT OF | 2021 | STATE WILDLIFE SURVEYS & INVESTIGATIONS - IDENTIFYING DEER POPULATION GENETIC STRUCTURE TO REDUCE THE SPREAD OF CHRONIC WASTING DISEASE | F20AF11583 | 1 |
| DOI, USFWS | $1,105,245 | GAME & FISH COMMISSION, ARKANSAS | 2021 | SUPPORT APPLIED RESEARCH TO PROVIDE NECESSARY INFORMATION THAT WILL ALLOW THE AGENCY TO PROACTIVELY MANAGE CWD TRANSMISSION AND PREVALENCE, WHILE ALSO UNDERSTANDING INDIVIDUAL AND POPULATION-LEVEL EFFECTS OF CWD. | F20AF00265 | 1 |
| DOI, USFWS | $244,946 | CORNELL UNIVERSITY | 2021 | SURVEILLANCE OPTIMIZATION PROJECT FOR CHRONIC WASTING DISEASE: STREAMLINING A WEB APPLICATION FOR DISEASE VISUALIZATION AND DATA-DRIVEN DECISIONS | F21AP00722 | 1 |
| DOI, USFWS | $136,313 | COLORADO STATE UNIVERSITY | 2021 | To deliver a coordinated and systematic approach for monitoring, investigating, and reporting on health problems in free-ranging wildlife throughout Colorado. Chronic Wasting Disease. | F21AF01562 | 1 |
| DOI, USFWS | $21,000 | NATURAL RESOURCES, IOWA DEPARTMENT OF | 2021 | WI-UPR MS RIV NATL WILDL AND FISH RFG-LA - CHRONIC WASTING DISEASE MONITORING | 140F0621P0287 | 1 |
| DOI, USGS | $207,197 | UNIVERSITY OF WISCONSIN | 2005 | FACTORS AFFECTING CWD TRANSMISSION | 03511HR099 | 1 |
| DOI, USGS | $93,250 | WISCONSIN DEPARTMENT OF NATURAL RESOURCES | 2006 | "COMPARATIVE STUDY OF TWO CHRONIC WASTING DISEASE (CWD) EPIDEMICS AS A FOUNDATION FOR ADAPTIVE DISEA | 06ERAG0010 | 1 |
| DOI, USGS | $40,000 | ILLINOIS DEPARTMENT OF NATURAL RESOURCES | 2007 | A COMPARATIVE STUDY OF TWO CWD EPIDEMICS AS A FOUNDATION FOR ADAPTIVE DISEASE MANAGEMENT STRATEGIES | 07ERAG0085 | 1 |
| DOI, USGS | $30,000 | UTAH STATE UNIVERSITY | 2008 | A LANDSCAPE GENETICS APPROACH TO ASSESSING CWD RISK IN UTAH | 06555HR057 | 1 |
| DOI, USGS | $124,288 | UNIVERISTY OF WYOMING | 2008 | EPIDEMIOLOGY OF CHRONIC WASTING DISEASE IN WHITE-TAILED DEAR... | 05542HR179 | 1 |
| DOI, USGS | $65,000 | UNIVERSITY OF ILLINOIS | 2008 | GENETICS AND GEOGRAPHY OF CHRONIC WASTING DISEASE... | 03075HS009 | 1 |
| DOI, USGS | $187,692 | UNIVERSITY OF WISCONSIN | 2008 | SPATIAL MODELING OF CHRONIC WASTING DISEASE EPIZOOTIOLOGY... | 03511HR109 | 1 |
| DOI, USGS | $520,280 | UNIVERSITY OF WYOMING | 2009 | POPULATION LEVEL IMPACTS OF CHRONIC WASTING DISEASE | G09AC00451 | 1 |
| DOI, USGS | $46,000 | UNIVERSITY OF WISCONSIN-MADISON | 2010 | HAS CWD PRODUCED GENETIC SELECTION IN THE WISCONSIN WHITE-TAILED DEER POPULATIONS? | G10AC00301 | 1 |
| DOI, USGS | $27,999 | VIRGINIA POLYTECHNIC INSTITUTE & ST TE UNIVER | 2012 | RESEARCH AND TECHNICAL ASSISTANCE FOR CHRONIC WASTING DISEASE SURVEILLANCE RESEARCH IN THE SHENANDOAH NATIONAL PARK | G12AC20478 | 1 |
| DOI, USGS | $76,754 | UNIVERSITY OF WYOMING | 2013 | POPULATION LEVEL IMPACTS OF CHRONIC WASTING DISEASE IN WYOMING MULE DEER II | G13AC00279 | 1 |
| DOI, USGS | $34,203 | UNIVERSITY OF WISCONSIN SYSTEM | 2014 | CWD DEPOSITION AND ENVIRONMENTAL RESERVOIRS | G14AC00355 | 1 |
| DOI, USGS | $52,613 | KANSAS STATE UNIVERSITY | 2018 | WHERE DO WE LOOK AND WHAT SHOULD WE DO: EXAMINING THE HARD QUESTIONS OF CHRONIC WASTING DISEASE MANAGEMENT AND SURVEILLANCE. | G18AC00317 | 1 |
| DOI, USGS | $24,833 | UNIVERSITY OF WISCONSIN SYSTEM | 2019 | ADVANCING IN VITRO PRION AMPLIFICATION ASSAYS FOR USE IN NON-INVASIVE AND ENVIRONMENTAL SAMPLING FOR DETECTION OF CHRONIC WASTING DISEASE | G19AC00394 | 1 |
| DOI, USGS | $52,612 | KANSAS STATE UNIVERSITY | 2019 | WHERE DO WE LOOK AND WHAT SHOULD WE DO: EXAMINING THE HARD QUESTIONS OF CHRONIC WASTING DISEASE MANAGEMENT AND SURVEILLANCE. | G18AC00317 | 1 |
| DOI, USGS | $642,555 | Various | 2019 | Additional USGS spending on CWD during fiscal year 2019 | Not applicable | 5 |
| DOI, USGS | $24,833 | UNIVERSITY OF WISCONSIN SYSTEM | 2020 | ADVANCING IN VITRO PRION AMPLIFICATION ASSAYS FOR USE IN NON-INVASIVE AND ENVIRONMENTAL SAMPLING FOR DETECTION OF CHRONIC WASTING DISEASE | G19AC00394 | 1 |
| DOI, USGS | $79,147 | BIO-RAD LABORATORIES, INC. | 2020 | CWD DIAGNOSTIC EQUIPMENT | 140G0220P0363 | 1 |
| DOI, USGS | $45,676 | UNIVERSITY OF WYOMING | 2020 | ENVIRONMENTAL PATHWAYS OF CHRONIC WASTING DISEASE TRANSMISSION | G20AC00399 | 1 |
| DOI, USGS | $199,265 | PENNSYLVANIA STATE UNIVERSITY, THE | 2020 | LINKING GENETICS TO MOVEMENTS OF WHITE-TAILED DEER TO ASSIST SURVEILLANCE FOR CHRONIC WASTING DISEASE | G20AC00460 | 1 |
| DOI, USGS | $67,680 | UNIVERSITY OF WISCONSIN SYSTEM | 2020 | MATHEMATICAL AND STATISTICAL MODELING AND FORECASTING OF CHRONIC WASTING DISEASE SPREAD | G20AC00126 | 1 |
| DOI, USGS | $70,621 | REGENTS OF THE UNIVERSITY OF MINNESOTA | 2020 | UNDERSTANDING PERCEPTIONS OF RISK FROM CHRONIC WASTING DISEASE FOR TRIBAL COMMUNITIES IN THE MIDWEST | G20AC00411 | 1 |
| DOI, USGS | $47,000 | KANSAS STATE UNIVERSITY | 2020 | WHERE DO WE LOOK AND WHAT SHOULD WE DO: EXAMINING THE HARD QUESTIONS OF CHRONIC WASTING DISEASE MANAGEMENT AND SURVEILLANCE. | G18AC00317 | 1 |
| DOI, USGS | $1,185,779 | Various | 2020 | Additional USGS spending on CWD during fiscal year 2020 | Not applicable | 5 |
| DOI, USGS | $208,500 | GAME AND FISH COMMISSION, WYOMING | 2021 | ASSESSING MINERAL LICKS AND PREDATION AS ALTERNATIVE MECHANISMS TO CONTROL CHRONIC WASTING DISEASE IN THE WEST | G21AC10127 | 1 |
| DOI, USGS | $18,264 | UTAH STATE UNIVERSITY | 2021 | CHRONIC WASTING DISEASE IN UTAH - DEVELOPMENT OF A RISK BASED CWD SURVEILLANCE SYSTEM WITH UPDATES FOR THE STATEWIDE CWD MANAGEMENT PLAN | G21AC10541 | 1 |
| DOI, USGS | $31,500 | FOUNDATIONS OF SUCCESS, INC. | 2021 | CROSS-BOUNDARY CHRONIC WASTING DISEASE MANAGEMENT WORKSHOP FACILITATION - FOUNDATION OF SUCCESS (FOS) | 140G0221P0184 | 1 |
| DOI, USGS | $125,549 | UNIVERSITY OF MONTANA | 2021 | DEVELOP BAYESIAN MODEL AND EVALUATION; COMPARISON OF MODELING APPROACHES AND COMPUTER SIMULATION FOR ESTIMATING DENSITY FROM REMOTE CAMERAS TO EXAMINE POPULATION IMPACT OF CWD | G21AC10546 | 1 |
| DOI, USGS | $45,676 | UNIVERSITY OF WYOMING | 2021 | ENVIRONMENTAL PATHWAYS OF CHRONIC WASTING DISEASE TRANSMISSION | G20AC00399 | 1 |
| DOI, USGS | $117,548 | PENNSYLVANIA STATE UNIVERSITY, THE | 2021 | ESTABLISHING A NATIONAL TISSUE AND REAGENTS REPOSITORY FOR CHRONIC WASTING DISEASE | G21AC10549 | 1 |
| DOI, USGS | $76,191 | UNIVERSITY OF WISCONSIN SYSTEM | 2021 | GOING UP IN SMOKE: ASSESSING THE ABILITY OF INCINERATION TO INACTIVE CWD PRIONS FROM CARCASSES | G21AC10486 | 1 |
| DOI, USGS | $85,229 | PENNSYLVANIA STATE UNIVERSITY, THE | 2021 | LINKING GENETICS TO MOVEMENTS OF WHITE-TAILED DEER TO ASSIST SURVEILLANCE FOR CHRONIC WASTING DISEASE | G20AC00460 | 1 |
| DOI, USGS | $75,930 | UNIVERSITY OF WISCONSIN SYSTEM | 2021 | MATHEMATICAL AND STATISTICAL MODELING AND FORECASTING OF CHRONIC WASTING DISEASE SPREAD | G20AC00126 | 1 |
| DOI, USGS | $63,041 | UTAH STATE UNIVERSITY | 2021 | MATHEMATICAL MODELS AND HOMOGENIZATION OF DEER DISPERSAL, ENVIRONMENTAL HAZARD, AND DIRECT/INDIRECT TRANSMISSION TO PREDICT SPREAD OF CHRONIC WASTING DISEASE | G21AC10553 | 1 |
| DOI, USGS | $2,872,572 | Various | 2021 | Additional USGS spending on CWD during fiscal year 2021 | Not applicable | 6 |
| HHS, NIH | $519,556 | COLORADO STATE UNIVERSITY-FORT COLLINS | 2000 | CWD: ROLE OF THE LYMPHOID TISSUE PHASE IN PRION DISEASE | K08AI001802 | 2 |
| HHS, NIH | $1,739,625 | COLORADO STATE UNIVERSITY-FORT COLLINS | 2000 | CWD--MODEL OF NVCJD AND LYMPHOID PATHOGENESIS IN TSE'S | 1R01NS040079-01 | 2 |
| HHS, NIH | $5,583,752 | UNIVERSITY OF KENTUCKY, COLORADO STATE UNIVERSITY | 2000 | Transgenetic studies of prion disease in cervids | R01NS040334 | 2 |
| HHS, NIH | $790,000 | DARTMOUTH COLLEGE | 2004 | Species Susceptibility Assay for Chronic Wasting Disease | R21AI058979 | 2 |
| HHS, NIH | $231,889 | WASHINGTON STATE UNIVERSITY | 2004 | Transmission of Prions Within and Between Species | K08AI060680 | 2 |
| HHS, NIH | $1,694,425 | CASE WESTERN RESERVE UNIVERSITY | 2006 | Assessing the Transmissibility of CWD to Humans | R01NS052319 | 2 |
| HHS, NIH | $12,804,034 | NATIONAL INSTITUTE OF ALLERGY AND INFECTIOUS DISEASES | 2007 | Study of CWD Deer and Elk Prion Disease in Nonhuman Primates | ZIAAI000983, Z01AI000983 | 2 |
| HHS, NIH | $2,108,552 | NATIONAL INSTITUTE OF ALLERGY AND INFECTIOUS DISEASES | 2007 | Study of TSE transmission and therapy | 1Z01AI000265 | 2 |
| HHS, NIH | $285,425 | SCRIPPS RESEARCH INSTITUTE | 2007 | Transmissible Spongiform Encephalopathy (Prion) Disease of Deer and Elk | R01NS053504 | 2 |
| HHS, NIH | $2,255,340 | UNIVERSITY OF WISCONSIN-MADISON | 2008 | Impact of Microparticles on Oral TSE Infections | R01NS060034 | 2 |
| HHS, NIH | $1,406,652 | COLORADO STATE UNIVERSITY | 2008 | Key molecular mechanisms of extraneural pathogenesis and transmission of TSEs | R01NS056379 | 2 |
| HHS, NIH | $3,018,822 | COLORADO STATE UNIVERSITY | 2009 | PMCA Detection of CWD Infection in Cervid and Non-Cervid Species | R01NS061902 | 2 |
| HHS, NIH | $219,482 | COLORADO STATE UNIVERSITY | 2010 | PATHOGENESIS OF CHRONIC WASTING DISEASE IN TRANSGENIC MICE | K01RR031488 | 1 |
| HHS, NIH | $327,457 | UNIVERSITY OF MINNESOTA | 2010 | PATHOGENESIS OF CHRONIC WASTING DISEASE IN TRANSGENIC MICE | K01OD010987 | 1 |
| HHS, NIH | $517,904 | COLORADO STATE UNIVERSITY, KANSAS STATE UNIVERSITY | 2010 | CWD: A Model of Prion Transmission via Saliva and Urine | K01OD010994, RR026270 | 2 |
| HHS, NIH | $2,196,790 | COLORADO STATE UNIVERSITY | 2011 | MOTHER TO OFFSPRING TRANSMISSION OF CHRONIC WASTING DISEASE | R01AI093634 | 1 |
| HHS, NIH | $3,158,949 | COLORADO STATE UNIVERSITY | 2014 | Detection and Characterization of Blood-borne Prions | R01AI112956 | 2 |
| HHS, NIH | $1,362,721 | CASE WESTERN RESERVE UNIVERSITY | 2015 | Cervid to human prion transmission | R01NS088604 | 2 |
| HHS, NIH | $176,405 | UNIVERSITY OF CALIFORNIA, SAN DIEGO | 2015 | Investigating molecular mechanisms of cross-species prion transmission | K01OD019919 | 2 |
| HHS, NIH | $3,180,800 | UNIVERSITY OF TEXAS HLTH SCI CTR HOUSTON | 2016 | CWD Prion Shedding and Environmental Contamination: Role in Transmission and Zoonotic Potential | Search by Project Title | 2 |
| HHS, NIH | $2,385,688 | UNIVERSITY OF TEXAS HLTH SCI CTR HOUSTON | 2016 | Zoonotic potential of CWD and influence of environmental contamination on prion propagation | Search by Project Title | 2 |
| HHS, NIH | $3,322,638 | UNIVERSITY OF TEXAS HEALTH SCIENCE CENTER AT HOUSTON, THE | 2017 | SHEDDING, RETENTION AND SPREADING OF CHRONIC WASTING DISEASE PRIONS IN THE ENVIRONMENT | R01AI132695 | 1 |
| HHS, NIH | $1,916,351 | COLORADO STATE UNIVERSITY | 2018 | CHARACTERIZING THE STRAIN AND HOST RANGE PROPERTIES OF PRIONS CAUSING EMERGENT FORMS OF CHRONIC WASTING DISEASE | R01NS109376 | 1 |
| HHS, NIH | $1,843,506 | COLORADO STATE UNIVERSITY | 2018 | Transmission and Pathogenesis of Chronic Wasting Disease (CWD) | R01NS061902 | 2 |
| HHS, NIH | $990,522 | COLORADO STATE UNIVERSITY | 2021 | ADDRESSING THE MECHANISMS OF PRION STRAIN EVOLUTION AND ITS EFFECT ON INTERSPECIES TRANSMISSION | R01NS121682 | 1 |
| HHS, NIH | $716,065 | COLORADO STATE UNIVERSITY | 2021 | CHRONIC WASTING DISEASE VACCINES | R01AI156037 | 1 |
| HHS, NIH | $270,000 | GOVERNORS OF THE UNIVERSITY OF CALGARY, THE | 2021 | REDEFINING THE ZOONOTIC POTENTIAL OF CHRONIC WASTING DISEASE | R01NS121016 | 1 |
| NASA | $346,000 | SENSIS INC. | 2020 | ANTEMORTEM ANIMAL TEST FOR THE EARLY DETECTION OF CHRONIC WASTING DISEASE (CWD) | 80JSC020F0330 | 1 |
| NSF | $2,217,878 | Colorado State Univ | 2000 | Spatial & Temporal Dynamics of Prion Disease in Wildlife: Responses to Changing Land Use | 0091961 | 3 |
| NSF | $2,499,881 | COLORADO STATE UNIVERSITY | 2009 | HIERARCHICAL BAYESIAN MODELING OF DISEASE DYNAMICS: A CASE EXAMPLE USING CHRONIC WASTING DISEASE | 914489 | 1 |
| NSF | $392,205 | UNIVERSITY OF WISCONSIN SYSTEM | 2009 | THE ROLE OF ENVIRONMENTAL AND DIRECT TRANSMISSION IN CHRONIC WASTING DISEASE DYNAMICS | 914484 | 1 |
| USDA, APHIS | $2,433,000 | Various | 2003 | Additional APHIS spending during fiscal year 2003 | Not applicable | 4 |
| **USDA, APHIS** | **$12,500,000** | **Various** | **2003** | **Indemnity payments** | **Not applicable** | **7** |
| USDA, APHIS | $18,522,000 | Various | 2004 | Additional APHIS spending during fiscal year 2004 | Not applicable | 4 |
| USDA, APHIS | $18,688,000 | Various | 2005 | Additional APHIS spending during fiscal year 2005 | Not applicable | 4 |
| USDA, APHIS | $18,523,000 | Various | 2006 | Additional APHIS spending during fiscal year 2006 | Not applicable | 4 |
| USDA, APHIS | $100,000 | KANSAS DEPARTMENT OF WILDLIFE AND PARKS | 2007 | MANAGING CHRONIC WASTING DISEASE IN FREE-RANGING CERVIDS | 08-9720-1657-CA | 1 |
| USDA, APHIS | $16,545,000 | Various | 2007 | Additional APHIS spending during fiscal year 2007 | Not applicable | 4 |
| USDA, APHIS | $35,030 | LOUISIANA DEPARTMENT OF WILDLIFE AND FISHERIE | 2008 | CHRONIC WASTING DISEASE - CWD SURVEILLANCE | 08-9722-1342-CA | 1 |
| USDA, APHIS | $245,000 | NEW YORK DEPARTMENT OF ENVIRONMENTAL CONSERVA | 2008 | CHRONIC WASTING DISEASE - FREE-RANGING CERVIDS - TO PROVIDE FEDERAL FINANCIAL ASSISTANCE FOR SURVEILLANCE AND TESTING | 08-9636-0201-CA | 1 |
| USDA, APHIS | $26,100 | NEVADA DEPARTMENT OF AGRICULTURE | 2008 | CHRONIC WASTING DISEASE (CWD) SURVEILLANCE | 08-9732-1716-CA | 1 |
| USDA, APHIS | $75,000 | IDAHO DEPARTMENT OF FISH AND GAME | 2008 | CHRONIC WASTING DISEASE (CWD) SURVEILLANCE ACTIVITIES | 08-9716-1666-CA | 1 |
| USDA, APHIS | $75,000 | MONTANA FISH, WILDLIFE AND PARKS DEPARTMENT | 2008 | CHRONIC WASTING DISEASE (CWD) SURVEILLANCE AND MANAGEMENT | 08-9730-1397-CA | 1 |
| USDA, APHIS | $270,567 | NATIVE AMERICAN FISH AND WILDLIFE SOCIETY | 2008 | CHRONIC WASTING DISEASE EDUCATION AND TESTING | 08-9108-1003-CA | 1 |
| USDA, APHIS | $285,000 | UTAH DIVISION OF WILDLIFE RESOURCES | 2008 | CHRONIC WASTING DISEASE MANAGEMENT AND SURVEILLANCE | 08-9749-1396-CA | 1 |
| USDA, APHIS | $75,000 | MISSOURI DEPARTMENT OF CONSERVATION | 2008 | CHRONIC WASTING DISEASE MANAGEMENT IN FREE RANGING CERVIDS | 08-9729-1643-CA | 1 |
| USDA, APHIS | $125,000 | UTAH DEPARTMENT OF AGRICULTURE AND FOOD | 2008 | CHRONIC WASTING DISEASE MANAGMENT AND SURVEILLANCE ACTIVITIES (EARMARK) | 08-9749-1395-CA | 1 |
| USDA, APHIS | $45,660 | CALIFORNIA DEPARTMENT OF FISH AND GAME | 2008 | CHRONIC WASTING DISEASE SURVEILLANCE | 08-9706-1713-CA | 1 |
| USDA, APHIS | $74,398 | TEXAS PARKS AND WILDLIFE | 2008 | CHRONIC WASTING DISEASE SURVEILLANCE | 08-9748-1679-CA | 1 |
| USDA, APHIS | $25,502 | NEW MEXICO DEPARTMENT OF GAME AND FISH | 2008 | CHRONIC WASTING DISEASE SURVEILLANCE ACTIVITIES | 08-9735-1697-CA | 1 |
| USDA, APHIS | $45,695 | NEVADA DEPARTMENT OF WILDLIFE | 2008 | CHRONIC WASTING DISEASE SURVEILLANCE ACTIVITIES | 08-9732-1721-CA | 1 |
| USDA, APHIS | $47,967 | ALASKA DEPARTMENT OF FISH AND GAME | 2008 | CHRONIC WASTING DISEASE SURVEILLANCE ACTIVITIES | 08-9702-1688-CA | 1 |
| USDA, APHIS | $48,000 | ARKANSAS GAME AND FISH COMMISSION | 2008 | CHRONIC WASTING DISEASE SURVEILLANCE ACTIVITIES | 08-9705-1706-CA | 1 |
| USDA, APHIS | $48,000 | WASHINGTON DEPARTMENT OF FISH AND WILDLIFE | 2008 | CHRONIC WASTING DISEASE SURVEILLANCE ACTIVITIES | 08-9753-1507-CA | 1 |
| USDA, APHIS | $75,000 | IOWA DEPARTMENT OF NATURAL RESOURCES | 2008 | CHRONIC WASTING DISEASE SURVEILLANCE ACTIVITIES | 08-9719-1391-CA | 1 |
| USDA, APHIS | $111,795 | SOUTH DAKOTA GAME, FISH AND PARKS | 2008 | CHRONIC WASTING DISEASE SURVEILLANCE ACTIVITIES | 08-9746-1680-CA | 1 |
| USDA, APHIS | $236,413 | NEBRASKA GAME AND PARKS COMMISSION | 2008 | CHRONIC WASTING DISEASE SURVEILLANCE ACTIVITIES | 08-9731-1387-CA | 1 |
| USDA, APHIS | $285,000 | WYOMING GAME AND FISH COMMISSION | 2008 | CHRONIC WASTING DISEASE SURVEILLANCE ACTIVITIES | 08-9756-1662-CA | 1 |
| USDA, APHIS | $60,400 | OKLAHOMA DEPARTMENT OF WILDLIFE CONSERVATION | 2008 | CHRONIC WASTING DISEASE SURVEILLANCE AND MANAGEMENT | 08-9740-1505-CA | 1 |
| USDA, APHIS | $75,000 | NORTH DAKOTA GAME AND FISH DEPARTMENT | 2008 | CHRONIC WASTING DISEASE SURVEILLANCE AND TESTING | 08-9738-1665-CA | 1 |
| USDA, APHIS | $285,000 | COLORADO DIVISION OF WILDLIFE | 2008 | CHRONIC WASTING DISEASE SURVEILLANCE. | 08-9708-1504-CA | 1 |
| USDA, APHIS | $54,440 | MARYLAND DEPARTMENT OF NATURAL RESOURCES | 2008 | CHRONIC WASTING DISEASE TESTING AND SURVEILLANCE IN THE STATE OF MARYLAND | 08-9624-0155-CA | 1 |
| USDA, APHIS | $285,000 | WISCONSIN DEPARTMENT OF NATURAL RESOURCES | 2008 | CHRONIC WASTING DISEASE: TO MINIMIZE THE NEGATIVE IMPACT OF CHRONIC WASTING DISEASE. | 08-9655-0224-CA | 1 |
| USDA, APHIS | $71,687 | INFORMATION TECHNOLOGY EXPERTS, INC. | 2008 | DEVELOPMENT OF A DISEASE MANAGEMENT SOFTWARE APPLICATION - CHRONIC WASTING DISEASE. | AG32KWP080271 | 1 |
| USDA, APHIS | $36,942 | UNIVERSITY OF WISCONSIN | 2008 | IMPACT OF FEEDING REGIMES ON THE POTENTIAL FOR DIRECT CHRONIC WASTING DISEASE (CWD) TRANSMISSION WITHIN AND AMONG MATERNAL GROUPS OF WHITE-TAI | 08-7488-0622-CA | 1 |
| USDA, APHIS | $194,710 | KANSAS DEPARTMENT OF WILDLIFE AND PARKS | 2008 | MANAGING CHRONIC WASTING DISEASE IN FREE-RANGING CERVIDS | 08-9720-1877-CA | 1 |
| USDA, APHIS | $32,945 | COLORADO STATE UNIVERSITY | 2008 | SUPPORT A PHD CANDIDATE WHO WILL CONDUCT RESEARCH RELATED TO NEW TECHNOLOGY IN DETECTING CHRONIC WASTING DISEASE. | 08-7488-0680-CA | 1 |
| USDA, APHIS | $138,015 | UNIVERSITY OF NEBRASKA | 2008 | SUPPORT OF CHRONIC WASTING DISEASE (CWD) STUDIES ASSOCIATED WITH DETERMINING CWD PREVALENCE, DEER DEMOGRAPHICS, AND MODELING THE SYSTEM. | 08-7488-0485-CA | 1 |
| USDA, APHIS | $73,371 | ARIZONA GAME AND FISH DEPARTMENT, GAME BRANCH | 2008 | SURVEILLANCE AND MANAGEMENT CHRONIC WASTING DISEASE | 08-9704-1693-CA | 1 |
| USDA, APHIS | $285,000 | ILLINOIS DEPARTMENT OF NATURAL RESOURCES | 2008 | TO SUPPORT CONTINUED SURVEILLANCE AND DISEASE MANAGEMENT OF CHRONIC WASTING DISEASE (CWD) IN FREE-RANGING CERVIDS. | 08-9617-0120-CA | 1 |
| USDA, APHIS | $13,834,363 | Various | 2008 | Additional APHIS spending during fiscal year 2008 | Not applicable | 4 |
| USDA, APHIS | $2,985 | HOPI TRIBE | 2009 | CHRONIC WASTING DISEASE | 09-9704-1538-CA | 1 |
| USDA, APHIS | $10,000 | COLORADO RIVER INDIAN TRIBE | 2009 | CHRONIC WASTING DISEASE | 09-9704-1837-CA | 1 |
| **USDA, APHIS** | **$13,110** | **ELK FARM LLC.** | **2009** | **CHRONIC WASTING DISEASE** | **10-3331-00118-IN** | **1** |
| **USDA, APHIS** | **$53,248** | **ELK FARM LLC.** | **2009** | **CHRONIC WASTING DISEASE** | **10-3331-00116-IN** | **1** |
| **USDA, APHIS** | **$666,544** | **ELK FARM LLC.** | **2009** | **CHRONIC WASTING DISEASE** | **10-3331-00117-IN** | **1** |
| USDA, APHIS | $44,550 | LOUISIANA DEPARTMENT OF WILDLIFE AND FISHERIE | 2009 | CHRONIC WASTING DISEASE - CWD SURVEILLANCE | 09-9722-1342-CA | 1 |
| USDA, APHIS | $7,563 | SHOSHONE-PAIUTE/DUCK VALLEY | 2009 | CHRONIC WASTING DISEASE (CWD) PROGRAM SURVEILLANCE | 09-9732-1889-CA | 1 |
| USDA, APHIS | $9,978 | NEVADA DEPARTMENT OF AGRICULTURE | 2009 | CHRONIC WASTING DISEASE (CWD) SURVEILLANCE | 09-9732-1716-CA | 1 |
| USDA, APHIS | $70,000 | MONTANA FISH, WILDLIFE AND PARKS DEPARTMENT | 2009 | CHRONIC WASTING DISEASE (CWD) SURVEILLANCE AND MANAGEMENT | 09-9730-1397-CA | 1 |
| USDA, APHIS | $248,120 | NATIVE AMERICAN FISH AND WILDLIFE SOCIETY | 2009 | CHRONIC WASTING DISEASE EDUCATION AND TESTING THE FUNDS GO TO THE NATIVE AMERICAN FISH AND WILDLIFE SOCIETY FOR USE WITH SEVERAL TRIBES | 09-9108-1003-CA | 1 |
| USDA, APHIS | $215,000 | UTAH DIVISION OF WILDLIFE RESOURCES | 2009 | CHRONIC WASTING DISEASE MANAGEMENT AND SURVEILLANCE | 09-9749-1396-CA | 1 |
| USDA, APHIS | $150,480 | UTAH DEPARTMENT OF AGRICULTURE AND FOOD | 2009 | CHRONIC WASTING DISEASE MANAGMENT AND SURVEILLANCE ACTIVITIES (EARMARK) | 09-9749-1395-CA | 1 |
| USDA, APHIS | $10,000 | WASHOE TRIBE OF NEVADA AND CALIFORNIA | 2009 | CHRONIC WASTING DISEASE SURVEILLANCE | 09-9732-1892-CA | 1 |
| USDA, APHIS | $10,000 | DUCKWATER SHOSHONE TRIBE | 2009 | CHRONIC WASTING DISEASE SURVEILLANCE | 09-9732-1972-CA | 1 |
| USDA, APHIS | $15,591 | CALIFORNIA DEPARTMENT OF FISH AND GAME | 2009 | CHRONIC WASTING DISEASE SURVEILLANCE | 09-9706-1713-CA | 1 |
| USDA, APHIS | $70,000 | TEXAS PARKS AND WILDLIFE | 2009 | CHRONIC WASTING DISEASE SURVEILLANCE | 09-9748-1679-CA | 1 |
| USDA, APHIS | $10,000 | YAKAMA NATION | 2009 | CHRONIC WASTING DISEASE SURVEILLANCE ACTIVITIES | 09-9753-1719-CA | 1 |
| USDA, APHIS | $21,641 | HAWAII DEPARTMENT OF LAND AND NATURAL RESOURC | 2009 | CHRONIC WASTING DISEASE SURVEILLANCE ACTIVITIES | 09-9715-1400-CA | 1 |
| USDA, APHIS | $45,000 | ARKANSAS GAME AND FISH COMMISSION | 2009 | CHRONIC WASTING DISEASE SURVEILLANCE ACTIVITIES | 09-9705-1706-CA | 1 |
| USDA, APHIS | $45,000 | WASHINGTON DEPARTMENT OF FISH AND WILDLIFE | 2009 | CHRONIC WASTING DISEASE SURVEILLANCE ACTIVITIES | 09-9753-1507-CA | 1 |
| USDA, APHIS | $59,973 | NEVADA DEPARTMENT OF WILDLIFE | 2009 | CHRONIC WASTING DISEASE SURVEILLANCE ACTIVITIES | 09-9732-1721-CA | 1 |
| USDA, APHIS | $70,000 | IOWA DEPARTMENT OF NATURAL RESOURCES | 2009 | CHRONIC WASTING DISEASE SURVEILLANCE ACTIVITIES | 09-9719-1391-CA | 1 |
| USDA, APHIS | $70,972 | NEW MEXICO DEPARTMENT OF GAME AND FISH | 2009 | CHRONIC WASTING DISEASE SURVEILLANCE ACTIVITIES | 09-9735-1697-CA | 1 |
| USDA, APHIS | $97,386 | SOUTH DAKOTA GAME, FISH AND PARKS | 2009 | CHRONIC WASTING DISEASE SURVEILLANCE ACTIVITIES | 09-9746-1680-CA | 1 |
| USDA, APHIS | $158,000 | NEBRASKA GAME AND PARKS COMMISSION | 2009 | CHRONIC WASTING DISEASE SURVEILLANCE ACTIVITIES | 09-9731-1387-CA | 1 |
| USDA, APHIS | $215,000 | WYOMING GAME AND FISH COMMISSION | 2009 | CHRONIC WASTING DISEASE SURVEILLANCE ACTIVITIES | 09-9756-1662-CA | 1 |
| USDA, APHIS | $10,000 | KAWERAK, INC. | 2009 | CHRONIC WASTING DISEASE SURVEILLANCE ACTIVITIES. | 09-9702-1691-CA | 1 |
| USDA, APHIS | $70,000 | NORTH DAKOTA GAME AND FISH DEPARTMENT | 2009 | CHRONIC WASTING DISEASE SURVEILLANCE AND EDUCATION | 09-9738-1665-CA | 1 |
| USDA, APHIS | $63,000 | OKLAHOMA DEPARTMENT OF WILDLIFE CONSERVATION | 2009 | CHRONIC WASTING DISEASE SURVEILLANCE AND MANAGEMENT | 09-9740-1505-CA | 1 |
| USDA, APHIS | $69,289 | ARIZONA GAME AND FISH DEPARTMENT, GAME BRANCH | 2009 | CHRONIC WASTING DISEASE SURVEILLANCE AND MANAGEMENT | 09-9704-1693-CA | 1 |
| USDA, APHIS | $10,000 | SAN CARLOS APACHE TRIBE | 2009 | CHRONIC WASTING DISEASE SURVEILLANCE AND OUTREACH | 09-9704-1880-CA | 1 |
| USDA, APHIS | $70,000 | MARYLAND DEPARTMENT OF NATURAL RESOURCES | 2009 | CHRONIC WASTING DISEASE TESTING AND SURVEILLANCE IN THE STATE OF MARYLAND | 09-9624-0155-CA | 1 |
| USDA, APHIS | $7,043 | MENOMINEE INDIAN TRIBE OF WISCONSIN | 2009 | CHRONIC WASTING DISEASE: FINANCIAL ASSISTANCE TO GENERATE INDIAN COUNTRY SURVEILLANCE, COMMUNICATION, AND INFORMATION DISSEMINATION CAPACITY IN AND | 09-9655-0317-CA | 1 |
| USDA, APHIS | $215,000 | WISCONSIN DEPARTMENT OF NATURAL RESOURCES | 2009 | CHRONIC WASTING DISEASE: THE GOALS OF THIS AGREEMENT ARE: 1) SURVEILLANCE; 2) HUMAN HEALTH PROTECTION; 3) RESEARCH; 4) COMMUNICATIONS; AND 5) DISE | 09-9655-0224-CA | 1 |
| USDA, APHIS | $31,680 | COLORADO STATE UNIVERSITY | 2009 | CONDUCT SURVEILLANCE ACTIVITIES FOR CHRONIC WASTING DISEASE (EARMARK) | 09-9708-1878-CA | 1 |
| USDA, APHIS | $8,337 | NAVAJO NATION | 2009 | CONDUCT SURVEILLANCE ACTIVITIES FOR THE CHRONIC WASTING DISEASE (CWD) PROGRAM | 09-9704-1958-CA | 1 |
| USDA, APHIS | $8,615 | ST. CROIX CHIPPEWA INDIANS OF WISCONSIN | 2009 | COOPERATIVE RELATIONSHIP BETWEEN THE ST. CROIX CHIPPEWA INDIANS AND APHIS FOR SURVEILLANCE OF CHRONIC WASTING DISEASE (CWD). | 09-9655-0996-CA | 1 |
| USDA, APHIS | $185,813 | KANSAS DEPARTMENT OF WILDLIFE AND PARKS | 2009 | MANAGING CHRONIC WASTING DISEASE IN FREE-RANGING CERVIDS | 09-9720-1877-CA | 1 |
| USDA, APHIS | $39,356 | COLORADO STATE UNIVERSITY | 2009 | SUPPORT A PHD CANDIDATE WHO WILL CONDUCT RESEARCH RELATED TO NEW TECHNOLOGY IN DETECTING CHRONIC WASTING DISEASE. | 09-7488-0680-CA | 1 |
| USDA, APHIS | $10,000 | UTAH DIVISION OF WILDLIFE RESOURCES | 2009 | THE UTAH DIVISION OF WILDLIFE RESOURCES 3RD INTERNATIONAL CHRONIC WASTING DISEASE SYMPOSIUM | 09-9149-1233-GR | 1 |
| USDA, APHIS | $194,000 | UNIVERSITY OF NEBRASKA | 2009 | TO CONDUCT RESEARCH THAT WILL PROVIDE INFORMATION ON THE POTENTIAL FOR AND RATE OF TRANSMISSION OF CHRONIC WASTING DISEASE IN WILD CERVIDS. | 09-7488-0485-CA | 1 |
| USDA, APHIS | $22,000 | DELAWARE DEPARTMENT OF NATURAL RESOURCES AND | 2009 | TO CONDUCT SURVEILLANCE ACTIVITIES FOR THE CHRONIC WASTING DISEASE (CWD) PROGRAM. | 09-9610-0153-CA | 1 |
| USDA, APHIS | $52,575 | OHIO DEPARTMENT OF AGRICULTURE | 2009 | TO CONDUCT SURVEILLANCE ACTIVITIES FOR THE CHRONIC WASTING DISEASE (CWD) PROGRAM. | 09-9639-0912-CA | 1 |
| USDA, APHIS | $502,128 | WISCONSIN DEPARTMENT OF NATURAL RESOURCES | 2009 | TO CONDUCT SURVEILLANCE ACTIVITIES FOR THE CHRONIC WASTING DISEASE (CWD) PROGRAM. | 09-9655-0381-CA | 1 |
| USDA, APHIS | $613,712 | WISCONSIN DEPARTMENT OF AGRICULTURE, TRADE AN | 2009 | TO CONDUCT SURVEILLANCE ACTIVITIES FOR THE CHRONIC WASTING DISEASE (CWD) PROGRAM. | 09-9655-0382-CA | 1 |
| USDA, APHIS | $10,000 | RED LAKE BAND OF CHIPPEWA INDIANS | 2009 | TO CONDUCT SURVEILLANCE ACTIVITIES FOR THE CHRONIC WASTING DISEASE PROGRAM. | 09-9627-0356-CA | 1 |
| USDA, APHIS | $215,000 | NEW YORK DEPARTMENT OF ENVIRONMENTAL CONSERVA | 2009 | TO CONDUCT SURVEILLANCE FOR CHRONIC WASTING DISEASE - FREE-RANGING CERVIDS | 09-9636-0201-CA | 1 |
| USDA, APHIS | $44,520 | NEW JERSEY DEPT. OF ENVIRONMENTAL PROTECTION, | 2009 | TO CONDUCT SURVEILLANCE FOR CHRONIC WASTING DISEASE (CWD). TIER 2 STATE. | 09-9634-0219-CA | 1 |
| USDA, APHIS | $9,976 | BAY MILLS INDIAN COMMUNITY | 2009 | TO CONDUCT SURVEILLANCE FOR CHRONIC WASTING DISEASE. | 09-9626-0999-CA | 1 |
| USDA, APHIS | $10,000 | KEWEENAW BAY INDIAN COMMUNITY | 2009 | TO CONDUCT SURVEILLANCE FOR CHRONIC WASTING DISEASE. | 09-9626-0819-CA | 1 |
| USDA, APHIS | $17,028 | UNIVERSITY OF NEBRASKA | 2009 | TO DETERMINE THE EFFICACY OF USING A DEVELOPED ENZYME TO DECONTAMINATE ENVIRONMENTAL SAMPLES THAT HAVE BEEN EXPOSED TO BOTH CHRONIC WASTING DISEASE | 09-7488-0711-CA | 1 |
| USDA, APHIS | $42,625 | MISSISSIPPI DEPARTMENT OF WILDLIFE, FISHERIES | 2009 | TO PROVIDE ASSISTANCE WITH SURVEILLANCE ACTIVITIES FOR THE CHRONIC WASTING DISEASE PROGRAM. | 09-9628-0156-CA | 1 |
| USDA, APHIS | $9,411 | MISSISSIPPI BAND OF CHOCTAW INDIANS | 2009 | TO PROVIDE FEDERAL FINANCIAL ASSISTANCE FOR THE CHRONIC WASTING DISEASE PROGRAM | 09-9628-0397-CA | 1 |
| USDA, APHIS | $10,000 | LEECH LAKE BAND OF OJIBWE | 2009 | TO PROVIDE FEDERAL FINANCIAL ASSISTANCE FOR THE CHRONIC WASTING DISEASE PROGRAM. | 09-9627-0907-CA | 1 |
| USDA, APHIS | $10,000 | WHITE EARTH RESERVATION BAND OF OJIBWE | 2009 | TO PROVIDE FEDERAL FINANCIAL ASSISTANCE FOR THE CHRONIC WASTING DISEASE PROGRAM. | 09-9627-0385-CA | 1 |
| USDA, APHIS | $70,000 | MINNESOTA DEPARTMENT OF NATURAL RESOURCES | 2009 | TO PROVIDE FEDERAL FINANCIAL ASSISTANCE FOR THE CHRONIC WASTING DISEASE PROGRAM. | 09-9627-0193-CA | 1 |
| USDA, APHIS | $70,000 | PENNSYLVANIA GAME COMMISSION | 2009 | TO PROVIDE FEDERAL FINANCIAL ASSISTANCE TO CONDUCT BOTH ACTIVE AND TARGETED SURVEILLANCE OF WILD DEER AND ELK FOR CHRONIC WASTING DISEASE (CWD) SURV | 09-9642-0310-CA | 1 |
| USDA, APHIS | $215,000 | ILLINOIS DEPARTMENT OF NATURAL RESOURCES | 2009 | TO SUPPORT CONTINUED SURVEILLANCE AND DISEASE MANAGEMENT OF CHRONIC WASTING DISEASE (CWD) IN FREE-RANGING CERVIDS. | 09-9617-0120-CA | 1 |
| USDA, APHIS | $11,311,790 | Various | 2009 | Additional APHIS spending during fiscal year 2009 | Not applicable | 4 |
| **USDA, APHIS** | **$31,112** | **LANDWER ROB** | **2010** | **CHRONIC WASTING DISEASE** | **10-3331-00205-IN** | **1** |
| USDA, APHIS | $7,913 | SHOSHONE-PAIUTE/DUCK VALLEY | 2010 | CHRONIC WASTING DISEASE (CWD) PROGRAM SURVEILLANCE | 10-9732-1889-CA | 1 |
| USDA, APHIS | $2,000 | FORT PECK ASSINIBOINE AND SIOUX TRIBES | 2010 | CHRONIC WASTING DISEASE (CWD) SURVEILLANCE AND OUTREACH/EDUCATION. | 10-9730-1883-CA | 1 |
| USDA, APHIS | $36,723 | LOUISIANA DEPARTMENT OF WILDLIFE AND FISHERIE | 2010 | CHRONIC WASTING DISEASE COMMUNICATION AND SURVEILLANCE ACTIVITIES | 10-9722-1342-CA | 1 |
| USDA, APHIS | $176,777 | KANSAS DEPARTMENT OF WILDLIFE AND PARKS | 2010 | CHRONIC WASTING DISEASE COMMUNICATION, DISEASE MANAGEMENT & SURVEILLANCE | 10-9720-1877-CA | 1 |
| USDA, APHIS | $7,604 | HOPI TRIBE | 2010 | CHRONIC WASTING DISEASE COMMUNICATION, DISEASE MANAGEMENT AND SURVEILLANCE | 10-9704-1538-CA | 1 |
| USDA, APHIS | $8,000 | TURTLE MOUNTAIN BAND OF CHIPPEWA | 2010 | CHRONIC WASTING DISEASE COMMUNICATION, INFO DISSEMINATION & SURVEILLANCE | 10-9738-2046-CA | 1 |
| USDA, APHIS | $66,117 | TEXAS PARKS AND WILDLIFE | 2010 | CHRONIC WASTING DISEASE COMMUNICATION, INFO DISSEMINATION, DISEASE MANAGEMENT & SURVEILLANCE | 10-9748-1679-CA | 1 |
| USDA, APHIS | $9,996 | NEVADA DEPARTMENT OF AGRICULTURE | 2010 | CHRONIC WASTING DISEASE COMMUNICATION, INFORMATION DISSEMINATION, DISEASE MANAGEMENT & SURVEILLANCE | 10-9732-1716-CA | 1 |
| USDA, APHIS | $53,515 | NEVADA DEPARTMENT OF WILDLIFE | 2010 | CHRONIC WASTING DISEASE COMMUNICATION, INFORMATION DISSEMINATION, DISEASE MANAGEMENT & SURVEILLANCE | 10-9732-1721-CA | 1 |
| USDA, APHIS | $55,787 | MINNESOTA BOARD OF ANIMAL HEALTH | 2010 | CHRONIC WASTING DISEASE CONTROL AND ERADICATION PROGRAM IN THE STATE OF MINNESOTA. | 10-9627-0811-CA | 1 |
| USDA, APHIS | $248,677 | NATIVE AMERICAN FISH AND WILDLIFE SOCIETY | 2010 | CHRONIC WASTING DISEASE EDUCATION AND TESTING THE FUNDS GO TO THE NATIVE AMERICAN FISH AND WILDLIFE SOCIETY FOR USE WITH SEVERAL TRIBES | 10-9108-1003-CA | 1 |
| USDA, APHIS | $260,000 | UTAH DIVISION OF WILDLIFE RESOURCES | 2010 | CHRONIC WASTING DISEASE MANAGEMENT AND SURVEILLANCE | 10-9749-1396-CA | 1 |
| USDA, APHIS | $10,000 | COLORADO RIVER INDIAN TRIBE | 2010 | CHRONIC WASTING DISEASE SURVEILLANCE | 10-9704-1837-CA | 1 |
| USDA, APHIS | $70,000 | ARIZONA GAME AND FISH DEPARTMENT, GAME BRANCH | 2010 | CHRONIC WASTING DISEASE SURVEILLANCE | 10-9704-1693-CA | 1 |
| USDA, APHIS | $10,000 | JEMEZ PUEBLO | 2010 | CHRONIC WASTING DISEASE SURVEILLANCE & TRAINING | 10-9735-2042-CA | 1 |
| USDA, APHIS | $10,000 | LOWER BRULE SIOUX TRIBE | 2010 | CHRONIC WASTING DISEASE SURVEILLANCE ACTIVITIES | 10-9746-1477-CA | 1 |
| USDA, APHIS | $44,996 | ALASKA DEPARTMENT OF FISH AND GAME | 2010 | CHRONIC WASTING DISEASE SURVEILLANCE ACTIVITIES | 09-9702-1688-CA | 1 |
| USDA, APHIS | $45,000 | WASHINGTON DEPARTMENT OF FISH AND WILDLIFE | 2010 | CHRONIC WASTING DISEASE SURVEILLANCE ACTIVITIES | 10-9753-1507-CA | 1 |
| USDA, APHIS | $70,000 | NORTH DAKOTA GAME AND FISH DEPARTMENT | 2010 | CHRONIC WASTING DISEASE SURVEILLANCE AND EDUCATION | 10-9738-1665-CA | 1 |
| USDA, APHIS | $143,496 | NEBRASKA GAME AND PARKS COMMISSION | 2010 | CHRONIC WASTING DISEASE SURVEILLANCE, COMMUNICATION & DISEASE MANAGEMENT ACTIVITIES | 10-9731-1387-CA | 1 |
| USDA, APHIS | $198,025 | COLORADO DIVISION OF WILDLIFE | 2010 | CHRONIC WASTING DISEASE SURVEILLANCE, DISEASE MANAGEMENT & EDUCATIONAL ACTIVITIES. | 10-9708-1504-CA | 1 |
| USDA, APHIS | $139,874 | COLORADO DIVISION OF WILDLIFE | 2010 | CHRONIC WASTING DISEASE SURVEILLANCE. | 09-9708-1504-CA | 1 |
| USDA, APHIS | $60,000 | MARYLAND DEPARTMENT OF NATURAL RESOURCES | 2010 | CHRONIC WASTING DISEASE TESTING AND SURVEILLANCE IN THE STATE OF MARYLAND | 10-9624-0155-CA | 1 |
| USDA, APHIS | $6,280 | MENOMINEE INDIAN TRIBE OF WISCONSIN | 2010 | CHRONIC WASTING DISEASE: FINANCIAL ASSISTANCE TO GENERATE INDIAN COUNTRY SURVEILLANCE, COMMUNICATION, AND INFORMATION DISSEMINATION CAPACITY | 10-9655-0317-CA | 1 |
| USDA, APHIS | $260,000 | WISCONSIN DEPARTMENT OF NATURAL RESOURCES | 2010 | CHRONIC WASTING DISEASE: THE GOALS OF THIS AGREEMENT ARE: 1) SURVEILLANCE; 2) HUMAN HEALTH PROTECTION; 3) RESEARCH; 4) COMMUNICATIONS; AND 5) DISE | 10-9655-0224-CA | 1 |
| USDA, APHIS | $5,443 | BLACKFEET TRIBE | 2010 | CONDUCT SURVEILLANCE ACTIVITIES FOR THE CHRONIC WASTING DISEASE (CWD) PROGRAM. | 10-9730-2055-CA | 1 |
| USDA, APHIS | $10,000 | YAKAMA NATION | 2010 | CONDUCT SURVEILLANCE ACTIVITIES FOR THE CHRONIC WASTING DISEASE (CWD) PROGRAM. | 10-9753-1719-CA | 1 |
| USDA, APHIS | $70,000 | MONTANA FISH, WILDLIFE AND PARKS DEPARTMENT | 2010 | CONDUCT SURVEILLANCE ACTIVITIES FOR THE CHRONIC WASTING DISEASE (CWD) PROGRAM. | 10-9730-1397-CA | 1 |
| USDA, APHIS | $215,000 | WYOMING GAME AND FISH COMMISSION | 2010 | CONDUCT SURVEILLANCE ACTIVITIES FOR THE CHRONIC WASTING DISEASE (CWD) PROGRAM. | 10-9756-1662-CA | 1 |
| USDA, APHIS | $34,955 | ARKANSAS GAME AND FISH COMMISSION | 2010 | CONDUCT SURVEILLANCE, TRAINING, COMMUNICATION,S AND DISEASE MANAGEMENT FOR THE CHRONIC WASTING DISEASE (CWD) PROGRAM. | 10-9705-1706-CA | 1 |
| USDA, APHIS | $10,000 | ST. CROIX CHIPPEWA INDIANS OF WISCONSIN | 2010 | COOPERATIVE RELATIONSHIP BETWEEN THE ST. CROIX CHIPPEWA INDIANS AND APHIS FOR SURVEILLANCE OF CHRONIC WASTING DISEASE (CWD). | 10-9655-0996-CA | 1 |
| USDA, APHIS | $10,000 | SAN CARLOS APACHE TRIBE | 2010 | OUTREACH & EDUCATION FOR THE CHRONIC WASTING DISEASE (CWD) PROGRAM | 10-9704-1880-CA | 1 |
| USDA, APHIS | $41,650 | COLORADO STATE UNIVERSITY - BOARD OF GOVERNOR | 2010 | SUPPORT A PHD CANDIDATE WHO WILL CONDUCT RESEARCH RELATED TO NEW TECHNOLOGY IN DETECTING CHRONIC WASTING DISEASE. | 10-7488-0680-CA | 1 |
| USDA, APHIS | $70,000 | MICHIGAN DEPARTMENT OF NATURAL RESOURCES | 2010 | TO CONDUCT BOTH ACTIVE AND TARGETED SURVEILLANCE OF WILD DEER AND ELK FOR CHRONIC WASTING DISEASE. | 10-9626-0194-CA | 1 |
| USDA, APHIS | $27,544 | UNIVERSITY OF NEBRASKA | 2010 | TO CONDUCT RESEARCH ON PRION INFECTIVITY AND DECONTAMINATION RELATIVE TO DIFFERING SOIL PARAMETERS AND LEVELS OF CHRONIC WASTING DISEASE CONTAMINATI | 10-7488-0711-CA | 1 |
| USDA, APHIS | $19,556 | DELAWARE DEPARTMENT OF NATURAL RESOURCES AND | 2010 | TO CONDUCT SURVEILLANCE ACTIVITIES FOR THE CHRONIC WASTING DISEASE (CWD) PROGRAM. | 10-9610-0153-CA | 1 |
| USDA, APHIS | $58,466 | OHIO DEPARTMENT OF AGRICULTURE | 2010 | TO CONDUCT SURVEILLANCE ACTIVITIES FOR THE CHRONIC WASTING DISEASE (CWD) PROGRAM. | 10-9639-0912-CA | 1 |
| USDA, APHIS | $405,504 | WISCONSIN DEPARTMENT OF NATURAL RESOURCES | 2010 | TO CONDUCT SURVEILLANCE ACTIVITIES FOR THE CHRONIC WASTING DISEASE (CWD) PROGRAM. | 10-9655-0381-CA | 1 |
| USDA, APHIS | $495,616 | WISCONSIN DEPARTMENT OF AGRICULTURE, TRADE AN | 2010 | TO CONDUCT SURVEILLANCE ACTIVITIES FOR THE CHRONIC WASTING DISEASE (CWD) PROGRAM. | 10-9655-0382-CA | 1 |
| USDA, APHIS | $10,000 | RED LAKE BAND OF CHIPPEWA INDIANS | 2010 | TO CONDUCT SURVEILLANCE ACTIVITIES FOR THE CHRONIC WASTING DISEASE PROGRAM. | 10-9627-0356-CA | 1 |
| USDA, APHIS | $45,015 | INDIANA DEPARTMENT OF NATURAL RESOURCES | 2010 | TO CONDUCT SURVEILLANCE ACTIVITIES THAT WILL PROVIDE INFORMATION FOR CHRONIC WASTING DISEASE (CWD). | 10-9618-0129-CA | 1 |
| USDA, APHIS | $9,199 | FOREST COUNTY POTAWATOMI | 2010 | TO CONDUCT SURVEILLANCE FOR CHRONIC WASTING DISEASE (CWD). | 10-9655-1070-CA | 1 |
| USDA, APHIS | $42,269 | NEW JERSEY DEPT. OF ENVIRONMENTAL PROTECTION, | 2010 | TO CONDUCT SURVEILLANCE FOR CHRONIC WASTING DISEASE (CWD). TIER 2 STATE. | 10-9634-0219-CA | 1 |
| USDA, APHIS | $9,993 | BAY MILLS INDIAN COMMUNITY | 2010 | TO CONDUCT SURVEILLANCE FOR CHRONIC WASTING DISEASE. | 10-9626-0999-CA | 1 |
| USDA, APHIS | $10,000 | KEWEENAW BAY INDIAN COMMUNITY | 2010 | TO CONDUCT SURVEILLANCE FOR CHRONIC WASTING DISEASE. | 10-9626-0819-CA | 1 |
| USDA, APHIS | $55,933 | MICHIGAN DEPARTMENT OF AGRICULTURE | 2010 | TO CONDUCT SURVEILLANCE FOR CHRONIC WASTING DISEASE. | 10-9626-1052-CA | 1 |
| USDA, APHIS | $68,225 | COLORADO STATE UNIVERSITY | 2010 | TO ESTABLISH A GREATER UNDERSTANDING OF THE TRANSMISSION OF CHRONIC WASTING DISEASE UTILIZING CAPTIVE DEER AND ELK. | 10-7488-0805-CA | 1 |
| USDA, APHIS | $28,426 | MISSISSIPPI DEPARTMENT OF WILDLIFE, FISHERIES | 2010 | TO PROVIDE ASSISTANCE WITH SURVEILLANCE ACTIVITIES FOR THE CHRONIC WASTING DISEASE PROGRAM. | 10-9628-0156-CA | 1 |
| USDA, APHIS | $9,999 | LEECH LAKE BAND OF OJIBWE | 2010 | TO PROVIDE FEDERAL FINANCIAL ASSISTANCE FOR THE CHRONIC WASTING DISEASE PROGRAM. | 10-9627-0907-CA | 1 |
| USDA, APHIS | $10,000 | WHITE EARTH RESERVATION BAND OF OJIBWE | 2010 | TO PROVIDE FEDERAL FINANCIAL ASSISTANCE FOR THE CHRONIC WASTING DISEASE PROGRAM. | 10-9627-0385-CA | 1 |
| USDA, APHIS | $70,000 | MINNESOTA DEPARTMENT OF NATURAL RESOURCES | 2010 | TO PROVIDE FEDERAL FINANCIAL ASSISTANCE FOR THE CHRONIC WASTING DISEASE PROGRAM. | 10-9627-0193-CA | 1 |
| USDA, APHIS | $70,000 | PENNSYLVANIA GAME COMMISSION | 2010 | TO PROVIDE FEDERAL FINANCIAL ASSISTANCE TO CONDUCT BOTH ACTIVE AND TARGETED SURVEILLANCE OF WILD DEER AND ELK FOR CHRONIC WASTING DISEASE (CWD) | 10-9642-0310-CA | 1 |
| USDA, APHIS | $211,091 | WEST VIRGINIA DIVISION OF NATURAL RESOURCES | 2010 | TO PROVIDE FEDERAL FINANCIAL ASSISTANCE TO CONDUCT SURVEILLANCE ACTIVITIES FOR THE CHRONIC WASTING DISEASE PROGRAM. | 09-9654-0152-CA | 1 |
| USDA, APHIS | $250,493 | WEST VIRGINIA DIVISION OF NATURAL RESOURCES | 2010 | TO PROVIDE FEDERAL FINANCIAL ASSISTANCE TO CONDUCT SURVEILLANCE ACTIVITIES FOR THE CHRONIC WASTING DISEASE PROGRAM. | 10-9654-0152-CA | 1 |
| USDA, APHIS | $260,000 | ILLINOIS DEPARTMENT OF NATURAL RESOURCES | 2010 | TO SUPPORT CONTINUED SURVEILLANCE AND DISEASE MANAGEMENT OF CHRONIC WASTING DISEASE (CWD) IN FREE-RANGING CERVIDS. | 10-9617-0120-CA | 1 |
| USDA, APHIS | $12,604,692 | Various | 2010 | Additional APHIS spending during fiscal year 2010 | Not applicable | 4 |
| **USDA, APHIS** | **$900** | **ALBIN, JEROME** | **2011** | **CHRONIC WASTING DISEASE** | **11-3331-00272-IN** | **1** |
| USDA, APHIS | $6,498 | BAND RIVER BAND OF LAKE SUPERIOR TRIBE OF CHI | 2011 | CHRONIC WASTING DISEASE (CWD) | 11-9655-1107-CA | 1 |
| USDA, APHIS | $10,120 | FORT PECK ASSINIBOINE AND SIOUX TRIBES | 2011 | CHRONIC WASTING DISEASE (CWD) SURVEILLANCE AND OUTREACH/EDUCATION. | 11-9730-1883-CA | 1 |
| USDA, APHIS | $160,147 | KANSAS DEPARTMENT OF WILDLIFE AND PARKS | 2011 | CHRONIC WASTING DISEASE COMMUNICATION, DISEASE MANAGEMENT & SURVEILLANCE | 11-9720-1877-CA | 1 |
| USDA, APHIS | $8,022 | HOPI TRIBE | 2011 | CHRONIC WASTING DISEASE COMMUNICATION, DISEASE MANAGEMENT AND SURVEILLANCE | 11-9704-1538-CA | 1 |
| USDA, APHIS | $10,000 | TURTLE MOUNTAIN BAND OF CHIPPEWA | 2011 | CHRONIC WASTING DISEASE COMMUNICATION, INFO DISSEMINATION & SURVEILLANCE | 11-9738-2046-CA | 1 |
| USDA, APHIS | $70,000 | TEXAS PARKS AND WILDLIFE | 2011 | CHRONIC WASTING DISEASE COMMUNICATION, INFO DISSEMINATION, DISEASE MANAGEMENT & SURVEILLANCE | 11-9748-1679-CA | 1 |
| USDA, APHIS | $155,514 | UTAH DIVISION OF WILDLIFE RESOURCES | 2011 | CHRONIC WASTING DISEASE COMMUNICATION, INFO DISSEMINATION, DISEASE MANAGEMENT & SURVEILLANCE ACTIVITIES | 11-9749-1396-CA | 1 |
| USDA, APHIS | $172,000 | NORTH DAKOTA GAME AND FISH DEPARTMENT | 2011 | CHRONIC WASTING DISEASE COMMUNICATION, INFO DISSEMINATION, DISEASE MANAGEMENT & SURVEILLANCE ACTIVITIES | 11-9738-1665-CA | 1 |
| USDA, APHIS | $61,687 | NEVADA DEPARTMENT OF WILDLIFE | 2011 | CHRONIC WASTING DISEASE COMMUNICATION, INFORMATION DISSEMINATION, DISEASE MANAGEMENT & SURVEILLANCE | 11-9732-1721-CA | 1 |
| USDA, APHIS | $140,000 | NATIVE AMERICAN FISH AND WILDLIFE SOCIETY | 2011 | CHRONIC WASTING DISEASE EDUCATION AND TESTING THE FUNDS GO TO THE NATIVE AMERICAN FISH AND WILDLIFE SOCIETY FOR USE WITH SEVERAL TRIBES | 11-9108-1003-CA | 1 |
| USDA, APHIS | $10,000 | LOWER BRULE SIOUX TRIBE | 2011 | CHRONIC WASTING DISEASE EDUCATION/OUTREACH AND SURVEILLANCE ACTIVITIES | 11-9746-1477-CA | 1 |
| USDA, APHIS | $5,277 | COLORADO RIVER INDIAN TRIBE | 2011 | CHRONIC WASTING DISEASE SURVEILLANCE | 11-9704-1837-CA | 1 |
| USDA, APHIS | $167,416 | COLORADO DIVISION OF WILDLIFE | 2011 | CHRONIC WASTING DISEASE SURVEILLANCE & INFO DISSEMINATION ACTIVITIES. | 11-9708-1504-CA | 1 |
| USDA, APHIS | $10,000 | JEMEZ PUEBLO | 2011 | CHRONIC WASTING DISEASE SURVEILLANCE & TRAINING ACTIVITIES | 11-9735-2042-CA | 1 |
| USDA, APHIS | $68,199 | ARIZONA GAME AND FISH DEPARTMENT, GAME BRANCH | 2011 | CHRONIC WASTING DISEASE SURVEILLANCE, COMMUNICATIONS, & INFORMATION DISSEMINATION | 11-9704-1693-CA | 1 |
| USDA, APHIS | $152,501 | MARYLAND DEPARTMENT OF NATURAL RESOURCES | 2011 | CHRONIC WASTING DISEASE TESTING AND SURVEILLANCE IN THE STATE OF MARYLAND | 11-9624-0155-CA | 1 |
| USDA, APHIS | $8,461 | MENOMINEE INDIAN TRIBE OF WISCONSIN | 2011 | CHRONIC WASTING DISEASE: FINANCIAL ASSISTANCE TO GENERATE INDIAN COUNTRY SURVEILLANCE, COMMUNICATION, AND INFORMATION DISSEMINATION CAPACITY | 11-9655-0317-CA | 1 |
| USDA, APHIS | $182,700 | WISCONSIN DEPARTMENT OF NATURAL RESOURCES | 2011 | CHRONIC WASTING DISEASE: THE GOALS OF THIS AGREEMENT ARE: 1) SURVEILLANCE; 2) HUMAN HEALTH PROTECTION; 3) RESEARCH; 4) COMMUNICATIONS; AND 5) DISE | 11-9655-0224-CA | 1 |
| USDA, APHIS | $6,223 | BLACKFEET TRIBE | 2011 | CONDUCT MONITORING & SURVEILLANCE ACTIVITIES FOR THE CHRONIC WASTING DISEASE (CWD) PROGRAM. | 11-9730-2055-CA | 1 |
| USDA, APHIS | $10,000 | YAKAMA NATION | 2011 | CONDUCT SURVEILLANCE ACTIVITIES FOR THE CHRONIC WASTING DISEASE (CWD) PROGRAM. | 11-9753-1719-CA | 1 |
| USDA, APHIS | $69,946 | MONTANA FISH, WILDLIFE AND PARKS DEPARTMENT | 2011 | CONDUCT SURVEILLANCE ACTIVITIES FOR THE CHRONIC WASTING DISEASE (CWD) PROGRAM. | 11-9730-1397-CA | 1 |
| USDA, APHIS | $172,000 | WYOMING GAME AND FISH COMMISSION | 2011 | CONDUCT SURVEILLANCE ACTIVITIES FOR THE CHRONIC WASTING DISEASE (CWD) PROGRAM. | 11-9756-1662-CA | 1 |
| USDA, APHIS | $10,000 | ST. CROIX CHIPPEWA INDIANS OF WISCONSIN | 2011 | COOPERATIVE RELATIONSHIP BETWEEN THE ST. CROIX CHIPPEWA INDIANS AND APHIS FOR SURVEILLANCE OF CHRONIC WASTING DISEASE (CWD). | 11-9655-0996-CA | 1 |
| USDA, APHIS | $10,000 | SAN CARLOS APACHE TRIBE | 2011 | OUTREACH & COMMUICATIONS FOR THE CHRONIC WASTING DISEASE (CWD) PROGRAM | 11-9704-1880-CA | 1 |
| USDA, APHIS | $75,634 | COLORADO STATE UNIVERSITY - BOARD OF GOVERNOR | 2011 | SUPPORT A PHD CANDIDATE WHO WILL CONDUCT RESEARCH RELATED TO NEW TECHNOLOGY IN DETECTING CHRONIC WASTING DISEASE. | 11-7488-0680-CA | 1 |
| USDA, APHIS | $70,000 | MICHIGAN DEPARTMENT OF NATURAL RESOURCES | 2011 | TO CONDUCT BOTH ACTIVE AND TARGETED SURVEILLANCE OF WILD DEER AND ELK FOR CHRONIC WASTING DISEASE. | 11-9626-0194-CA | 1 |
| USDA, APHIS | $22,000 | DELAWARE DEPARTMENT OF NATURAL RESOURCES AND | 2011 | TO CONDUCT SURVEILLANCE ACTIVITIES FOR THE CHRONIC WASTING DISEASE (CWD) PROGRAM. | 11-9610-0153-CA | 1 |
| USDA, APHIS | $53,447 | OHIO DEPARTMENT OF AGRICULTURE | 2011 | TO CONDUCT SURVEILLANCE ACTIVITIES FOR THE CHRONIC WASTING DISEASE (CWD) PROGRAM. | 11-9639-0912-CA | 1 |
| USDA, APHIS | $10,000 | RED LAKE BAND OF CHIPPEWA INDIANS | 2011 | TO CONDUCT SURVEILLANCE ACTIVITIES FOR THE CHRONIC WASTING DISEASE PROGRAM. | 11-9627-0356-CA | 1 |
| USDA, APHIS | $46,680 | INDIANA DEPARTMENT OF NATURAL RESOURCES | 2011 | TO CONDUCT SURVEILLANCE ACTIVITIES THAT WILL PROVIDE INFORMATION FOR CHRONIC WASTING DISEASE (CWD). | 11-9618-0129-CA | 1 |
| USDA, APHIS | $6,453 | FOREST COUNTY POTAWATOMI | 2011 | TO CONDUCT SURVEILLANCE FOR CHRONIC WASTING DISEASE (CWD). | 11-9655-1070-CA | 1 |
| USDA, APHIS | $25,898 | NEW JERSEY DEPT. OF ENVIRONMENTAL PROTECTION, | 2011 | TO CONDUCT SURVEILLANCE FOR CHRONIC WASTING DISEASE (CWD). TIER 2 STATE. | 11-9634-0219-CA | 1 |
| USDA, APHIS | $9,416 | BAY MILLS INDIAN COMMUNITY | 2011 | TO CONDUCT SURVEILLANCE FOR CHRONIC WASTING DISEASE. | 11-9626-0999-CA | 1 |
| USDA, APHIS | $10,000 | KEWEENAW BAY INDIAN COMMUNITY | 2011 | TO CONDUCT SURVEILLANCE FOR CHRONIC WASTING DISEASE. | 11-9626-0819-CA | 1 |
| USDA, APHIS | $19,712 | MISSISSIPPI DEPARTMENT OF WILDLIFE, FISHERIES | 2011 | TO PROVIDE ASSISTANCE WITH SURVEILLANCE ACTIVITIES FOR THE CHRONIC WASTING DISEASE PROGRAM. | 11-9628-0156-CA | 1 |
| USDA, APHIS | $9,999 | LEECH LAKE BAND OF OJIBWE | 2011 | TO PROVIDE FEDERAL FINANCIAL ASSISTANCE FOR THE CHRONIC WASTING DISEASE PROGRAM. | 11-9627-0907-CA | 1 |
| USDA, APHIS | $10,000 | WHITE EARTH RESERVATION BAND OF OJIBWE | 2011 | TO PROVIDE FEDERAL FINANCIAL ASSISTANCE FOR THE CHRONIC WASTING DISEASE PROGRAM. | 11-9627-0385-CA | 1 |
| USDA, APHIS | $182,700 | MINNESOTA DEPARTMENT OF NATURAL RESOURCES | 2011 | TO PROVIDE FEDERAL FINANCIAL ASSISTANCE FOR THE CHRONIC WASTING DISEASE PROGRAM. | 11-9627-0193-CA | 1 |
| USDA, APHIS | $70,000 | PENNSYLVANIA GAME COMMISSION | 2011 | TO PROVIDE FEDERAL FINANCIAL ASSISTANCE TO CONDUCT BOTH ACTIVE AND TARGETED SURVEILLANCE OF WILD DEER AND ELK FOR CHRONIC WASTING DISEASE (CWD) | 11-9642-0310-CA | 1 |
| USDA, APHIS | $176,327 | WEST VIRGINIA DIVISION OF NATURAL RESOURCES | 2011 | TO PROVIDE FEDERAL FINANCIAL ASSISTANCE TO CONDUCT SURVEILLANCE ACTIVITIES FOR THE CHRONIC WASTING DISEASE PROGRAM. | 11-9654-0152-CA | 1 |
| USDA, APHIS | $182,700 | ILLINOIS DEPARTMENT OF NATURAL RESOURCES | 2011 | TO SUPPORT CONTINUED SURVEILLANCE AND DISEASE MANAGEMENT OF CHRONIC WASTING DISEASE (CWD) IN FREE-RANGING CERVIDS. | 11-9617-0120-CA | 1 |
| USDA, APHIS | $14,117,423 | Various | 2011 | Additional APHIS spending during fiscal year 2011 | Not applicable | 4 |
| USDA, APHIS | $3,687,000 | Various | 2012 | Additional APHIS spending during fiscal year 2012 | Not applicable | 4 |
| USDA, APHIS | $2,802,000 | Various | 2013 | Additional APHIS spending during fiscal year 2013 | Not applicable | 4 |
| **USDA, APHIS** | **$41,237** | **LEE, JAKE** | **2014** | **CHRONIC WASTING DISEASE** | **14-1909-782657-I** | **1** |
| USDA, APHIS | $65,000 | COLORADO STATE UNIVERSITY | 2014 | THE PURPOSE OF THIS AGREEMENT IS TO RESEARCH THE DEVELOPMENT OF AN ANTE MORTEM CHRONIC WASTING DISEASE ASSAY. | 14-7488-1105-CA | 1 |
| USDA, APHIS | $4,503,763 | Various | 2014 | Additional APHIS spending during fiscal year 2014 | Not applicable | 4 |
| **USDA, APHIS** | **$2,209** | **YODER, ERVIN** | **2015** | **CHRONIC WASTING DISEASE** | **15-1912-515261-I** | **1** |
| **USDA, APHIS** | **$2,850** | **SCHMIDT, TYLER** | **2015** | **CHRONIC WASTING DISEASE** | **16-1915-434542-I** | **1** |
| **USDA, APHIS** | **$3,000** | **SIMON, DARYL** | **2015** | **CHRONIC WASTING DISEASE** | **15-1913-497875-I** | **1** |
| **USDA, APHIS** | **$3,000** | **RICHTER, ROBERT** | **2015** | **CHRONIC WASTING DISEASE** | **16-1915-344868-I** | **1** |
| **USDA, APHIS** | **$6,000** | **UTLEY, RUSSELL** | **2015** | **CHRONIC WASTING DISEASE** | **15-1912-396908-I** | **1** |
| **USDA, APHIS** | **$19,451** | **DAY, DERIK** | **2015** | **CHRONIC WASTING DISEASE** | **15-1912-570093-I** | **1** |
| **USDA, APHIS** | **$400,000** | **PATTERSON, ROBERT** | **2015** | **CHRONIC WASTING DISEASE** | **16-1915-015944-I** | **1** |
| **USDA, APHIS** | **$583,813** | **YODER, DANIEL** | **2015** | **CHRONIC WASTING DISEASE** | **15-1913-525405-I** | **1** |
| USDA, APHIS | $854,721 | Various | 2015 | Additional APHIS spending during fiscal year 2015 | Not applicable | 4 |
| **USDA, APHIS** | **$1,875** | **MAST, MARK** | **2016** | **CHRONIC WASTING DISEASE** | **16-1917-883915-I** | **1** |
| **USDA, APHIS** | **$2,541** | **MILLER, LEROY** | **2016** | **CHRONIC WASTING DISEASE** | **15-1912-514839-I** | **1** |
| **USDA, APHIS** | **$3,000** | **OLSON, PERRY** | **2016** | **CHRONIC WASTING DISEASE** | **15-1913-653996-I** | **1** |
| **USDA, APHIS** | **$6,500** | **MAST, MARK** | **2016** | **CHRONIC WASTING DISEASE** | **16-1918-333778-I** | **1** |
| **USDA, APHIS** | **$8,750** | **HERSHBERGER, ANDY** | **2016** | **CHRONIC WASTING DISEASE** | **16-1918-828953-I** | **1** |
| **USDA, APHIS** | **$11,500** | **YODER, DAVID** | **2016** | **CHRONIC WASTING DISEASE** | **16-1916-808072-I** | **1** |
| **USDA, APHIS** | **$16,000** | **TROYER, NORMAN** | **2016** | **CHRONIC WASTING DISEASE** | **16-1918-915376-I** | **1** |
| **USDA, APHIS** | **$36,000** | **MILLER, LEROY** | **2016** | **CHRONIC WASTING DISEASE** | **16-1917-771116-I** | **1** |
| **USDA, APHIS** | **$39,000** | **MILLER, LEROY** | **2016** | **CHRONIC WASTING DISEASE** | **16-1918-657774-I** | **1** |
| **USDA, APHIS** | **$104,395** | **NOONER, SAMMY** | **2016** | **CHRONIC WASTING DISEASE** | **16-1917-824198-I** | **1** |
| **USDA, APHIS** | **$119,415** | **HOWE, QUINN** | **2016** | **CHRONIC WASTING DISEASE** | **15-1912-396909-I** | **1** |
| USDA, APHIS | $1,775,980 | Various | 2016 | Additional APHIS spending during fiscal year 2016 | Not applicable | 4 |
| USDA, APHIS | $2,000,000 | Various | 2017 | Additional APHIS spending during fiscal year 2017 | Not applicable | 4 |
| USDA, APHIS | $2,000,000 | Various | 2018 | Additional APHIS spending during fiscal year 2018 | Not applicable | 4 |
| USDA, APHIS | $21,991 | IOWA STATE UNIVERSITY OF SCIENCE AN | 2019 | CHRONIC WASTING DISEASE IN FERAL SWINE IN ARKANSAS | AP19WSHQ0000C001 | 1 |
| USDA, APHIS | $2,478,009 | Various | 2019 | Additional APHIS spending during fiscal year 2019 | Not applicable | 4 |
| USDA, APHIS | $241,512 | UNIVERSITY OF TEXAS HEALTH SCIENCE CENTER AT HOUSTON, THE | 2020 | Assessment of Protein Misfolding Cyclic Amplification (PMCA) and real-time quaking induced conversion (RT-QuIC) for Chronic Wasting Disease (CWD) diagnosis in animal and environmental samples | AP20VSSPRS00C143 | 1 |
| **USDA, APHIS** | **$2,850** | **RUDY BEACHY** | **2020** | **INDEMNITY-CWD-BEACHY, RUDY-00GW7VS** | **AP20MRPBSFOY0198** | **1** |
| **USDA, APHIS** | **$76,500** | **WATER DISPOSAL INC** | **2020** | **INDEMNITY-CWD-ROYAL VELVET RANCH-004CHEA** | **AP20MRPBSFOY0204** | **1** |
| **USDA, APHIS** | **$7,500** | **SAGEWOOD LIVESTOCK LLC** | **2020** | **INDEMNITY-CWD-SAGEWOOD LIVESTOCK LLC-JONES,OTTO-003TLKI** | **AP20MRPBSFOY0199** | **1** |
| **USDA, APHIS** | **$102,000** | **MARK W SIPES** | **2020** | **INDEMNITY-CWD-SIPES, MARK-BATTLE RIDGE WHITETAILS-003J16L** | **AP20MRPBSFOY0203** | **1** |
| **USDA, APHIS** | **$48,888** | **ELMER J STOLTZFUS** | **2020** | **INDEMNITY-CWD-STOLTZFUS, ELMER- 003L1LG** | **AP20MRPBSFOY0200** | **1** |
| **USDA, APHIS** | **$6,000** | **NORMAN A TROYER** | **2020** | **INDEMNITY-CWD-TROYER, NORMAN-00JTC7Y** | **AP21MRPBSFOY0033** | **1** |
| **USDA, APHIS** | **$8,550** | **MARK VOLK** | **2020** | **INDEMNITY-CWD-VOLK, MARK-MN324091** | **AP21MRPBSFOY0030** | **1** |
| **USDA, APHIS** | **$2,850** | **KENNETH WILLIAMS** | **2020** | **INDEMNITY-CWD-WILLIAMS, KENNETH-MN140508** | **AP21MRPBSFOY0029** | **1** |
| **USDA, APHIS** | **$2,850** | **JACOB YODER** | **2020** | **INDEMNITY-CWD-YODER, JAKE 00JGVBD** | **AP21MRPBSFOY0018** | **1** |
| **USDA, APHIS** | **$2,850** | **QUINN MUSCH** | **2020** | **INDEMNITY-IND-CWD-MUSCH, QUINN-00NEGZK** | **AP20MRPBSFOY0192** | **1** |
| USDA, APHIS | $181,207 | WILDLIFE RESOURCES AGENCY, TENNESSE | 2020 | TRAINED CANINE DETECTION OF CHRONIC WASTING DISEASE INFECTION | AP20VSSPRS00C130 | 1 |
| USDA, APHIS | $9,977,693 | Various | 2020 | Additional APHIS spending during fiscal year 2020 | Not applicable | 4 |
| USDA, APHIS | $166,177 | GRAND PORTAGE RESERVATION TRIBAL CO | 2021 | CHRONIC WASTING DISEASE SURVEILLANCE IN MIDWESTERN INDIAN CONTRY, DEVELOPMENT OF A REGIONAL SURVEILLANCE SYSTEM TO PROTECT TRIBAL SUBSISTENCE SPECIES. | AP20VSSPRS00C142 | 1 |
| USDA, APHIS | $249,987 | COLORADO STATE UNIVERSITY | 2021 | ENHANCEMENT OF EXISTING AND DEVELOPMENT OF NOVEL DIAGNOSTIC TOOLS FOR CHRONIC WASTING DISEASE. | AP21WSNWRC00C052 | 1 |
| USDA, APHIS | $12,481 | AGRICULTURE, NEBRASKA DEPARTMENT OF | 2021 | EQUINE CERVID SMALL RUMINANT EQUINE INITIATIVES - FY21 PROVIED TO CONTROL AND PREVENT CHRONIC WASTING DISEASE IN FARMEDCERVIDS ACTIVITIES. | AP21VSSPRS00C123 | 1 |
| USDA, APHIS | $429,598 | ANIMAL HEALTH COMMISSION, TEXAS | 2021 | EQUINE CERVID SMALL RUMINANT INITIATIVES - FY21 DEPOPULATIONOF A CHRONIC WASTING DISEASE -POSITIVE HERD IN UVALDE COUNTY, TEXAS ACTIVITIES | AP21VSSPRS00C128 | 1 |
| USDA, APHIS | $64,190 | MICHIGAN DEPARTMENT OF NATURAL RESO | 2021 | GENETIC IMPLICATIONS IN CHRONIC WASTING DISEASE MANAGEMENT O MICHIGAN WHITE-TAILED DEER | AP20VSSPRS00C141 | 1 |
| USDA, APHIS | $449,717 | MISSISSIPPI STATE UNIVERSITY | 2021 | GREATER PREVALENCE RATES OF CHRONIC WASTING DISEASE (CWD) IN MALE WHITE-TAILED DEER SUGGEST GENDER-SPECIFIC BEHAVIORS MAY FACILITATE DISEASE SPREAD (KINSELL 2010). | AP21WSNWRC00C044 | 1 |
| USDA, APHIS | $81,956 | UTAH STATE UNIVERSITY | 2021 | Improving surveillance of Chronic Wasting Disease (CWD) in UTah. | AP20VSSPRS00C134 | 1 |
| **USDA, APHIS** | **$5,700** | **JAMIE FEIST** | **2021** | **IND-CHRONIC WASTING DISEASE-FEIST, JAMIE-NO PREMISE ID** | **AP21MRPBSFOY0166** | **1** |
| **USDA, APHIS** | **$5,700** | **MARK VOLK** | **2021** | **IND-CHRONIC WASTING DISEASE-VOLK, MARK-PREMISE ID-MN324091** | **AP21MRPBSFOY0129** | **1** |
| **USDA, APHIS** | **$2,850** | **WASSERLOCH** | **2021** | **INDEMNITY ANIMAL HEALTH/DISEASE CONTROL DISEASE: CHRONIC WASTING DISEASE REIMBURSE PRODUCER FOR LOSS WASSERLOCH FARMER/PRODUCER NO SUBRECIPIENTS** | **AP22MRPBSFOY0009** | **1** |
| **USDA, APHIS** | **$3,800** | **ROBERT GERTEN** | **2021** | **INDEMNITY-CHRONIC WASTING DISEASE GERTEN, BOB-PREMISE ID-00PGHSN** | **AP21MRPBSFOY0164** | **1** |
| **USDA, APHIS** | **$143,038** | **PEK HEATING & AIR LLC** | **2021** | **INDEMNITY-CHRONIC WASTING DISEASE -PEK HEATING & AIR LLC-NATAN LUTZ-PREMISE ID 00P7DSX** | **AP21MRPBSFOY0154** | **1** |
| **USDA, APHIS** | **$23,700** | **BRAD SUESS** | **2021** | **INDEMNITY-CHRONIC WASTING DISEASE -SUESS, BRAD-PREMIS ID-MN53437** | **AP21MRPBSFOY0128** | **1** |
| **USDA, APHIS** | **$470,304** | **MICHAEL L WOODS** | **2021** | **INDEMNITY-CHRONIC WASTING DISEASE -WOODS, MICHAEL-PREMISE IDFID1718B** | **AP21MRPBSFOY0123** | **1** |
| **USDA, APHIS** | **$45,000** | **AUTUMN ANTLERS INC** | **2021** | **INDEMNITY-CHRONIC WASTING DISEASE-AUTUMN ANTLERS-PREMISE IDMN143722** | **AP21MRPBSFOY0124** | **1** |
| **USDA, APHIS** | **$215,168** | **BARKLEY YOUNG** | **2021** | **INDEMNITY-CHRONIC WASTING DISEASE-BARKLEY YOUNG-PREMISE ID 03FZLX** | **AP21MRPBSFOY0099** | **1** |
| **USDA, APHIS** | **$209,994** | **JUSTIN E BURCH** | **2021** | **INDEMNITY-CHRONIC WASTING DISEASE-BURCH, JUSTIN-PREMISE ID 0QSGKY** | **AP21MRPBSFOY0155** | **1** |
| **USDA, APHIS** | **$17,100** | **CAMP FREEDOM INC** | **2021** | **INDEMNITY--CHRONIC WASTING DISEASE-CAMP FREEDOM-BACHENBERG,WLLIAM-PREMIS ID 00NW6LK** | **AP21MRPBSFOY0137** | **1** |
| **USDA, APHIS** | **$23,462** | **DEAN PAGE** | **2021** | **INDEMNITY-CHRONIC WASTING DISEASE-CW-PAGE, DEAN-PREMISE ID M-140592** | **AP21MRPBSFOY0075** | **1** |
| **USDA, APHIS** | **$2,850** | **EDDIE GINGERICH** | **2021** | **INDEMNITY-CHRONIC WASTING DISEASE-GINGERICH, EDDIE-PREMISE I-00DHKKK** | **AP21MRPBSFOY0153** | **1** |
| **USDA, APHIS** | **$5,850** | **LUKE HANSON** | **2021** | **INDEMNITY-CHRONIC WASTING DISEASE-HANSON, LUKE-PREMISE ID MS05130** | **AP21MRPBSFOY0131** | **1** |
| **USDA, APHIS** | **$29,969** | **HOWARD JOHNSON** | **2021** | **INDEMNITY-CHRONIC WASTING DISEASE-HOWARD JOHNSON-PREMISE ID0GW7VS** | **AP21MRPBSFOY0074** | **1** |
| **USDA, APHIS** | **$3,000** | **MICHAEL KRETCHMER** | **2021** | **INDEMNITY-CHRONIC WASTING DISEASE-KRESTCHMER, MIKE-PREMISE I 00Q1FEF** | **AP21MRPBSFOY0156** | **1** |
| **USDA, APHIS** | **$5,375** | **NEEDLES VIEW RANCH LLC** | **2021** | **INDEMNITY-CHRONIC WASTING DISEASE-NEEDLES VIEW RANCH,REYELTSDAVE-NO PREMISE ID** | **AP21MRPBSFOY0152** | **1** |
| **USDA, APHIS** | **$5,700** | **BRUCE PAPPAS** | **2021** | **INDEMNITY-CHRONIC WASTING DISEASE-PAPPAS, BRUCE -NORWAY RIDG WHITETAILS-NO PREMISE ID JULY 2021** | **AP21MRPBSFOY0127** | **1** |
| **USDA, APHIS** | **$40,631** | **ROBERT SCHULTZ** | **2021** | **INDEMNITY-CHRONIC WASTING DISEASE-ROBERT SCHULTZ-NO PREMISED 03/03/21** | **AP21MRPBSFOY0071** | **1** |
| **USDA, APHIS** | **$145,356** | **DEAN PAGE** | **2021** | **INDEMNITY-CHRONIC WASTING DISEASES-DEAN, PAGE- PREMISE ID-MN40592** | **AP21MRPBSFOY0112** | **1** |
| **USDA, APHIS** | **$51,000** | **TRIPLE D GAME FARM** | **2021** | **INDEMNITY-CHRONIC WASTING DISEASE-TRIPLE D GAME FARM-DEIST-PEMISE ID-00MW2B2** | **AP21MRPBSFOY0163** | **1** |
| **USDA, APHIS** | **$2,850** | **KENNETH WILLIAMS** | **2021** | **INDEMNITY-CHRONIC WASTING DISEASE-WILLIAMS-KENNETH-PREMISE I-MN140508** | **AP21MRPBSFOY0125** | **1** |
| **USDA, APHIS** | **$6,000** | **ANTLERS FROM THE WOODS** | **2021** | **INDEMNITY-CWD-ANTLERS FROM THE WOOD-00NUW7Y** | **AP21MRPBSFOY0041** | **1** |
| **USDA, APHIS** | **$20,427** | **CHASE FORNENGO** | **2021** | **INDEMNITY-CWD-FORNENGO, CHASE-00M3GDQ** | **AP20MRPBSFOY0193** | **1** |
| **USDA, APHIS** | **$116,575** | **JOSHUA WOODARD** | **2021** | **INDEMNITY-CWD-JOSHUA AND MIKE WOODARD-MN143709** | **AP21MRPBSFOY0057** | **1** |
| USDA, APHIS | $178,277 | UNIVERSITY OF TEXAS HEALTH SCIENCE | 2021 | TEST SAMPLES VIA PROTEIN MISFOLDING CYCLIC AMPLIFICATION (PMCA) IN ATOTAL OF 100 ANIMALS FROM CHRONIC WASTING DISEASE ENDEMIC REGIONS INTEXAS. | AP22WSHQ0000C001 | 1 |
| USDA, APHIS | $246,461 | MICHIGAN DEPARTMENT OF NATURAL RESO | 2021 | WS PROTECTION OF NATURAL RESOURCES INITIATIVE; A MULTICENTERVALIDATION OF A REAL-TIME QUAKING INDUCED CONVERSION ASSAY FOR SENSITIVE DETECTION OF CHRONIC WASTING DISEASE | AP21WSNWRC00C042 | 1 |
| USDA, APHIS | $75,451 | FISH AND GAME, MASSACHUSETTS DEPART | 2021 | WS PROTECTION OF NATURAL RESOURCES INITIATIVE; A RISK ASSESSENT OF CHRONIC WASTING DISEASE IN MASSACHUSETTS AND THE PROACTIVE DEVELOPMENT OF A COMMUNICATION STRATEGY | AP21WSNWRC00C016 | 1 |
| USDA, APHIS | $101,646 | FISH & WILDLIFE CONSERVATION COMMIS | 2021 | WS PROTECTION OF NATURAL RESOURCES INITIATIVE; APPLYING ASSOIATION OF FISH AND WILDLIFE AGENCIES BEST MANAGEMENT PRACTICES FOR CHRONIC WASTING DISEASE RISK ASSESSMENT AND SURVEILLANCE IN FLORIDA | AP21WSNWRC00C017 | 1 |
| USDA, APHIS | $90,000 | GAME & FISH COMMISSION, ARKANSAS | 2021 | WS PROTECTION OF NATURAL RESOURCES INITIATIVE; ARKANSAS GAMEAND FISH COMMISSION CHRONIC WASTING DISEASE TESTING AWARENESS AND OUTREACH PROMOTION | AP21WSNWRC00C028 | 1 |
| USDA, APHIS | $16,659 | FORT BELKNAP INDIAN COMMUNITY | 2021 | WS PROTECTION OF NATURAL RESOURCES INITIATIVE; CHRONIC WASTING DISEASE SURVEILLANCE ON FORT BELKNAP | AP21WSNWRC00C035 | 1 |
| USDA, APHIS | $49,760 | NATURAL RESOURCES WEST VA DIV | 2021 | WS PROTECTION OF NATURAL RESOURCES INITIATIVE; HUNTER ATTITUES TOWARD CHRONIC WASTING DISEASE AND MANAGEMENT EFFORTS INA TWO-COUNTY DISEASE-POSITIVE AREA IN WEST VIRGINIA | AP21WSNWRC00C038 | 1 |
| USDA, APHIS | $98,112 | KANSAS DEPARTMENT OF WILDLIFE, PARK | 2021 | WS PROTECTION OF NATURAL RESOURCES INITIATIVE; IMPLEMENTING COMPREHENSIVE MOLECULAR METHODS PACKAGE FOR CHRONIC WASTING DISEASE SURVEILLANCE THROUGH KANSAS | AP21WSNWRC00C024 | 1 |
| USDA, APHIS | $129,822 | WILDLIFE, NEVADA DEPARTMENT OF | 2021 | WS PROTECTION OF NATURAL RESOURCES INITIATIVE; IMPROVING HUNER PARTICIPATION AND AWARENESS FOR CHRONIC WASTING DISEASE IN NEVADA | AP21WSNWRC00C020 | 1 |
| USDA, APHIS | $203,829 | NATURAL RESOURCES, IOWA DEPARTMENT | 2021 | WS PROTECTION OF NATURAL RESOURCES INITIATIVE; LEVERAGING INENTIVIZED HARVEST, LOCAL HUNTER-LANDOWNER PARTNERSHIPS, ANDTESTING WITH AMPLIFICATION ASSAYS TO SLOW THE SPREAD OF CHRONIC WASTING DISEASE | AP21WSNWRC00C039 | 1 |
| USDA, APHIS | $94,986 | NATURAL RESOURCES, WISCONSIN DEPT O | 2021 | WS PROTECTION OF NATURAL RESOURCES INITIATIVE; MOTIVATING BEAVIOR CHANGE TOWARD CHRONIC WASTING DISEASE BEST PRACTICES THOUGH IMPROVED OUTREACH AND EDUCATION; WISCONSIN | AP21WSNWRC00C029 | 1 |
| USDA, APHIS | $47,023 | VIRGINIA DEPARTMENT OF WILDLIFE RES | 2021 | WS PROTECTION OF NATURAL RESOURCES INITIATIVE; OPTIMIZATIONF CHRONIC WASTING DISEASE SURVEILLANCE AND DATA MANAGEMENT STRATEGIES IN VIRGINIA | AP21WSNWRC00C022 | 1 |
| USDA, APHIS | $26,925 | VIRGINIA DEPARTMENT OF WILDLIFE RES | 2021 | WS PROTECTION OF NATURAL RESOURCES INITIATIVE; PERCEIVED RIS, BEHAVIORS, AND ATTITUDES RELATED TO CHRONIC WASTING DISEASE IN VIRGINIA | AP21WSNWRC00C021 | 1 |
| USDA, APHIS | $98,781 | NORTHWEST INDIAN FISHERIES COMMISSI | 2021 | WS PROTECTION OF NATURAL RESOURCES INITIATIVE; PREPARING WESERN WASHINGTON TREATY TRIBES FOR CHRONIC WASTING DISEASE THROUGH EDUCATIONAL PROGRAMS, WORKSHOPS AND SURVEILLANCE PROGRAMS. | AP21WSNWRC00C026 | 1 |
| USDA, APHIS | $126,000 | NATURAL RESOURCES, WISCONSIN DEPT O | 2021 | WS PROTECTION OF NATURAL RESOURCES INITIATIVE; REDUCING RISK OF CHRONIC WASTING DISEASE TRANSMISSION THROUGH FACILITATING PROPER CARCASS DISPOSAL; WISCONSIN | AP21WSNWRC00C025 | 1 |
| USDA, APHIS | $149,890 | NATURAL RESOURCES, MINNESOTA DEPART | 2021 | WS PROTECTION OF NATURAL RESOURCES INITIATIVE; SURVEILLANCEOR CHRONIC WASTING DISEASE IN WILD DEER SURROUNDING A RECENTLY DETECTED CHRONIC WASTING DISEASE-POSITIVE CAPTIVE CERVIDFACILITY | AP21WSNWRC00C040 | 1 |
| USDA, APHIS | $212,500 | WILDLIFE RESOURCES AGENCY, TENNESSE | 2021 | WS PROTECTION OF NATURAL RESOURCES INITIATIVE; TRAINED CANIN DETECTION OF FECAL SAMPLE ODOR AND WHOLE-BODY ODOR ASSOCIATED WITH CHRONIC WASTING DISEASE INFECTION IN WHITE-TAILED DEER AND CERVIDS | AP21WSNWRC00C019 | 1 |
| USDA, APHIS | $10,340,223 | Various | 2021 | Additional APHIS spending during fiscal year 2021 | Not applicable | 4 |
| USDA, APHIS | $274,968 | ANIMAL HEALTH COMMISSION, TEXAS | 2022 | EQUINE CERVID SMALL RUMINANT - FY21 DEPOPULATION OF A CHRONIC WASTING DISEASE -POSITIVE HERD IN UVALDE COUNTY, GONZALEZ WHITETAIL DEER RANCH UVALDE, TEXAS AND GONZALEZ LEVEL 5, REGION 4 ACTIVITIES | AP21VSSPRS00C134 | 1 |
| USDA, APHIS | $47,933 | NATURAL RESOURCES, INDIANA DEPARTME | 2022 | WS PROTECTION OF NATURAL RESOURCES INITIATIVE; ENZYME-LINKEDIMMUNOSORBENT ASSAY TESTING IN INDIANA; SUPPORTS CHRONIC WASTING DISEASE SURVEILLANCE, RESPONSE AND MANAGEMENT ACTIVITIES | AP21WSNWRC00C041 | 1 |
| USDA, ARS | $30,000 | UNIVERSITY OF KENTUCKY RESEARCH FOUNDATION | 2006 | TRANSGENIC ANALYSIS OF CHRONIC WASTING DISEASE STRAINS | 58-5348-6-131-2 | 1 |
| USDA, ARS | $55,999 | UNIVERSITY OF WASHINGTON | 2007 | STRAIN TYPING OF CHRONIC WASTING DISEASE (CWD) AND SCRAPIE BY INTRACEREBRAL INOCULATION INTO TRANSGENIC AND INBRED MOUSE LINES | 58-5348-7-466-6 | 1 |
| USDA, ARS | $100,000 | UNIVERSITY OF WASHINGTON | 2007 | STRAIN TYPING OF CHRONIC WASTING DISEASE (CWD) AND SCRAPIE BY INTRACEREBRAL INOCULATION INTO TRANSGENIC AND INBRED MOUSE LINES | 58-5348-7-466-2 | 1 |
| USDA, ARS | $150,000 | UNIVERSITY OF WASHINGTON | 2007 | STRAIN TYPING OF CHRONIC WASTING DISEASE (CWD) AND SCRAPIE BY INTRACEREBRAL INOCULATION INTO TRANSGENIC AND INBRED MOUSE LINES | 58-5348-7-466-5 | 1 |
| USDA, ARS | $36,756 | IOWA STATE UNIVERSITY OF SCIENCE AND TECHNOLOGY | 2018 | SKIN TEST TO DETECT CHRONIC WASTING DISEASE INFECTION | 5850308073 | 1 |
| USDA, NIFA | -$25,000 | UNIVERSITY OF WYOMING | 2008 | GENE DISCOVERY IN PLAGUE, BRUCELLOSIS AND TULAREMIA; HOST-PATHOGEN INTERACTIONS AND IMMUNE RESPONSE IN CHRONIC WASTING DISEASE | 2.00834E+13 | 1 |
| USDA, NIFA | $499,527 | UNIVERSITY OF TENNESSEE | 2021 | ECONOMIC IMPLICATIONS OF CHRONIC WASTING DISEASE (CWD) ON DEER HUNTING | 2.02167E+13 | 1 |
